# Estradiol-mediated inhibition of Sp1 decreases miR-3194-5p expression to enhance CD44 expression during lung cancer progression

**DOI:** 10.1186/s12929-022-00787-1

**Published:** 2022-01-17

**Authors:** Ming-Jer Young, Yung-Ching Chen, Shao-An Wang, Hui-Ping Chang, Wen-Bin Yang, Chia-Chi Lee, Chia-Yu Liu, Yau-Lin Tseng, Yi-Ching Wang, H. Sunny Sun, Wen-Chang Chang, Jan-Jong Hung

**Affiliations:** 1 Department of Biotechnology and Bioindustry Sciences, National Cheng Kung University, Tainan, Taiwan; 2 Division of Thoracic Surgery, Department of Surgery, College of Medicine National, Cheng Kung University, Tainan, Taiwan; 3 Department of Pharmacology, College of Medicine, National Cheng Kung University, Tainan, Taiwan; 4 The Ph.D. Program for Neural Regenerative Medicine, College of Medical Science and Technology, Taipei Medical University, Taipei, Taiwan; 5 Graduate Institute of Medical Sciences, College of Medicine, Taipei Medical University, Taipei, Taiwan; 6 School of Respiratory Therapy, College of Medicine, Taipei Medical University, Taipei, Taiwan; 7 Institute of Molecular Medicine, College of Medicine, National Cheng Kung University, Tainan, Taiwan; 8 TMU Research Center of Neuroscience, Taipei Medical University, 11031 Taipei, Taiwan

**Keywords:** Sp1, Estradiol (E2), microRNAs, CD44, RNF4, Lung cancer

## Abstract

**Background:**

Sp1, an important transcription factor, is involved in the progression of various cancers. Our previous studies have indicated that Sp1 levels are increased in the early stage of lung cancer progression but decrease during the late stage, leading to poor prognosis. In addition, estrogen has been shown to be involved in lung cancer progression. According to previous studies, Sp1 can interact with the estrogen receptor (ER) to coregulate gene expression. The role of interaction between Sp1 and ER in lung cancer progression is still unknown and will be clarified in this study.

**Methods:**

The clinical relevance between Sp1 levels and survival rates in young women with lung cancer was studied by immunohistochemistry. We validated the sex dependence of lung cancer progression in EGFR^L858R^-induced lung cancer mice. Wound healing assays, chamber assays and sphere formation assays in A549 cells, Taxol-induced drug-resistant A549 (A549-T24) and estradiol (E2)-treated A549 (E2-A549) cells were performed to investigate the roles of Taxol and E2 in lung cancer progression. Luciferase reporter assays, immunoblot and q-PCR were performed to evaluate the interaction between Sp1, microRNAs and CD44. Tail vein-injected xenograft experiments were performed to study lung metastasis. Samples obtained from lung cancer patients were used to study the mRNA level of CD44 by q-PCR and the protein levels of Sp1 and CD44 by immunoblot and immunohistochemistry.

**Results:**

In this study, we found that Sp1 expression was decreased in premenopausal women with late-stage lung cancer, resulting in a poor prognosis. Tumor formation was more substantial in female EGFR^L858R^ mice than in male mice and ovariectomized female mice, indicating that E2 might be involved in the poor prognosis of lung cancer. We herein report that Sp1 negatively regulates metastasis and cancer stemness in E2-A549 and A549-T24 cells. Furthermore, E2 increases the mRNA and protein levels of RING finger protein 4 (RNF4), which is the E3-ligase of Sp1, and thereby decreases Sp1 levels by promoting Sp1 degradation. Sp1 can be recruited to the promoter of miR-3194-5p, and positively regulate its expression. Furthermore, there was a strong inverse correlation between Sp1 and CD44 levels in clinical lung cancer specimens. Sp1 inhibited CD44 expression by increasing the expression of miR-3194-5p, miR-218-5p, miR-193-5p, miR-182-5p and miR-135-5p, ultimately resulting in lung cancer malignancy.

**Conclusion:**

Premenopausal women with lung cancer and decreased Sp1 levels have a poor prognosis. E2 increases RNF4 expression to repress Sp1 levels in premenopausal women with lung cancer, thus decreasing the expression of several miRNAs that can target CD44 and ultimately leading to cancer malignancy.

**Supplementary Information:**

The online version contains supplementary material available at 10.1186/s12929-022-00787-1.

## Background

Lung cancer, including non-small-cell lung cancer (NSCLC) and small cell lung cancer (SCLC), is the leading cause of cancer-related death worldwide. Approximately 85% of all new cases are NSCLC, and adenocarcinoma is the most common subtype of NSCLC. In the past three decades, the lung cancer incidence rate has decreased approximately twofold faster in men than in women. Furthermore, the lung cancer mortality rate has decreased in men but increased in women [1–3]. According to previous studies, up to 53% of women but only 15% of men who develop NSCLC have never smoked, indicating that other factors influence the development of NSCLC in women [4, 5]. In addition, the clinical characteristics of men and women are very different. Previous studies have indicated that when female patients are analyzed based on hormonal status, premenopausal women have a worse prognosis than both men and postmenopausal women, suggesting that estrogen promotes lung cancer malignancy in premenopausal women [6]. However, the detailed mechanism(s) remain to be elucidated. Recent studies have indicated that some Asian women with lung cancer have a poor prognosis [7, 8], and this is a very difficult and complicated issue because it is related to many possible factors such as race and gender. Gender differences include chromosome differences, genetic polymorphisms, sex steroid repertoires and levels, reproductive organs, metabolism and immune responses, all of which play a crucial role during cancer tumorigenesis and malignancy [9]. Gender also involves behaviors and activities related to differences in society and culture. According to previous studies, lung cancer is recognized as a different disease in women and men [10]. A detail understanding of why lung cancer in women leads to poor outcomes is very important and will be beneficial for the prevention of lung cancer in women [11].

CD44, as a critical biomarker of stem cells, has been reported to be involved in epithelial mesenchymal transition (EMT) during cancer progression [12]. To date, twelve isoforms of CD44 have been reported by the alternative splicing under different physiological or pathological conditions [12–14]. Only the short form of CD44, CD44st, has a different 3’UTR; the others, including the standard form, CD44s, have the same 3′UTR [15]. CD44 activity is regulated by various posttranslational modifications, such as glycosylation and phosphorylation [14]. CD44 can interact with hyaluronic acid (HA) to regulate cell migration, proliferation and differentiation by regulating signal transduction and gene expression [16]. In addition to the transcriptional activity, alternative splicing and 3′UTR regulation of CD44 expression and isoform types, CD44 can also be cleaved in the cytosolic fraction to release the CD44 intracellular domain (ICD), and then it is transferred into the nucleus to regulate genes expression involved in EMT and cancer stemness [17].

Sp1, as an important transcription factor, is involved in many cellular processes, including cell cycle progression, apoptosis, differentiation and tumorigenesis, by regulating the expression of many genes, including coding and noncoding genes [18–20]. The expression of Sp1 is higher in most cancer cells and tissues than in normal cells and tissues. Sp1 is tightly regulated in the early and late stages of tumorigenesis to influence cancer progression [21]. Studies in lung cancer cohorts have indicated that Sp1 is upregulated in most patients with early-stage lung cancer and promotes cancer growth [19, 21]. However, in late-stage lung cancer, a low level of Sp1 is correlated with a poor prognosis [21, 22], but the detailed mechanism remains unknown. Previous studies have also indicated that estrogen receptor β (ER-β) in the lung can positively regulate lung cancer progression. Several reports have also shown that Sp1 and ER can coregulate gene expression [23–26], implying that Sp1 and estrogen receptors coregulate lung cancer progression in women. In addition, previous studies have indicated that posttranslational modification of Sp1 is important for regulating its transactivity and protein degradation, which are involved in physiological and pathological conditions such as cancer progression. For example, Sp1 phosphorylation by JNK1/2 kinases increases protein stability [27]. Sp1 sumoylation at Lys16 increases Sp1 degradation [28]. In this study, we found that the increase in Sp1 degradation in premenopausal women with lung cancer can inhibit Sp1-mediated miRNA expression, thus activating EMT-related gene expression and leading to lung cancer malignancy.

## Methods

### Cells culture and reagents

Human lung adenocarcinoma epithelial cell lines, A549 and H1299, were cultured with RPMI 1640 medium (Corning, New York, NY, USA) containing 10% fetal bovine serum (FBS) (Gibco, Invitrogen, NY, USA), 100 μg/ml streptomycin and 100 U/ml penicillin G. A549-T24 cells were obtained by exposing A549 cells to stepwise increased dose of Taxol until 24 nM (Sigma Aldrich, St. Louis, MO, USA). E2-A549 cells were obtained by treating A549 cells with 1 μg/ml estradiol (E2) for 3 weeks. All cells were maintained in a humidified incubator under 5% CO2 at 37 °C. Trichostatin A (TSA) and suberoylanilide hydroxamic acid (SAHA) were purchased from Cayman Chemical (Ann Arbor, MI, USA).

### Lentivirus-based shRNA expression

The lenti-scramble, lenti-Sp1-shRNA and lenti-CD44-shRNA viruses were generated from the National RNAi Core Facility (Academia Sinica, Taipei, Taiwan). Cells were seeded in 6-well-plates and incubated for 16 h, and then treated with 1 ml RPMI medium containing 10 μg polybrene (Merck Millipore, Darmstadt, Germany) and lentivirus with 5 multiplicity of infection (MOI). After 24 h of infection, medium containing lentivirus was replaced with fresh medium and maintained for another 72 h.

### Recombinant adenovirus

A adenovirus carrying the DNA encoding green fluorescent protein (GFP) or GFP-Sp1. Adenoviruses, adeno-GFP and adeno-GFP-Sp1, were prepared as previously described [29]. A549, A549-T24 and E2-A549 cells were infected with Sp1-overexpressing adenovirus or control adenovirus (MOI = 20) for 48 h.

### Transduction of lentiviral microRNA sponge constructs

The lentiviruses carrying miR-3194/324/218/200/193/182/135 and control sponges were generated from the National RNAi Core Facility (Academia Sinica). For transduction, the lentiviral miRNA sponges supplemented with 8 µg/ml polybrene were added to the A549-T24 and E2-A549 cells seeded at 5.0 × 10^5^ cells/ well for 24 h.

### Immunohistochemistry (IHC)

Human and mouse specimens were incubated in 10% formaldehyde for 72 h for fixation, dehydration, and embedded in paraffin. For immunohistochemistry, xylene was used for dewaxing paraffin-embedded sections and serial diluted ethanol was also used for dehydration. Endogenous peroxidases were blocked by incubating in PBS containing 0.3% hydrogen peroxide for 30 min, and then samples were blocked with 1% bovine serum albumin. Proteins of interest were recognized by incubated with anti-Sp1 (Merck Millipore, 1:500), anti-ALDH1 (Cat# 611,194, BD Biosciences, San Jose, CA, USA, 1:500), anti-CD44 (Cat# 102,111, GeneTex, 1:250), anti-vimentin (Cat# 5741, Cell Signaling, 1:50) and anti-β-catenin antibodies (Cat# 8480, Cell Signaling, 1:200) at room temperature for 3 h, and immunoreactivity was visualized by using Vectastain ABC kit (Vector Laboratories, Burlingame, CA, USA). Sections were photographed by Olympus BX-51 microscope (Olympus Corporation, Tokyo, Japan).

### Animal care and transgenic mice

TetO-EGFR^L858R^ transgenic mice expressing EGFR^L858R^ are regulated by Scgb1a1 regulated tetracycline-responsive promoter element (TRE; tet-on) (Jackson Laboratory, Bar Harbor, ME, USA). To induce EGFR^L858R^ expression in the lung of transgenic mice, doxycycline (0.5 g/l) was added to drinking water from 8 weeks of age. In addition, the in vivo metastasis assay was performed by using severe combined immunodeficiency (SCID) mice purchased from the Animal Center of National Cheng Kung University. 1 × 10^6^ A549 and E2-A549 cells with or without GFP-Sp1 overexpression were prepared and suspended in PBS, then tumor cells were injected into 8-week-old female SCID mice through tail vein. Five weeks after injection, mice were sacrificed, and metastatic lung area were counted by ImageJ. Tumor burden was calculated by the lung area of the induced mice subtracted the lung area of normal mice.

### Ovariectomy surgeries

Mice with 8 weeks of age underwent either ovariectomy or sham surgery under anesthesia induced by intraperitoneal injection of ketamine (100 mg/kg) and xylazine (7 mg/kg). Ovariectomy surgeries involved bilateral flank incisions through the skin and muscle wall and the removal of ovaries. Sham surgeries involved bilateral flank incisions through the skin and muscle wall. Incisions were closed using sterile 5–0 ETHILON nylon (NC125, UNIC, Taiwan) sutures. Nalbuphine (5 mg/kg) (Uni Pharma, Taiwan) was administered by subcutaneous injection before the start of each surgery. Mice were single housed following surgery. E2 concentrations were quantified using a commercially available EIA kit (Arbor Assays, Ann Arbor, MI, USA).

### RT-PCR and q-PCR

Total RNA from cell lines was isolated using Direct-zol RNA MiniPrep Plus kit (Zymo Research, Irvine, CA, USA) according to the vending company's instructions. RNA was subsequently reverse-transcribed with a Mir-XTM miRNA q-PCR TB Green Kit (#638,314, TAKARA, Otsu, Japan). The cDNA was used as a template for quantitative real-time PCR analysis using KAPA SYBR FAST qPCR Kit Master Mix (2 ×) (#KK4601, KAPA Biosystems, Wilmington, MA, USA). All values were normalized with internal control, glyceraldehyde 3-phosphate dehydrogenase (GAPDH), and relative gene expression levels were then calculated. Because the endogenous U6 gene is generally used to normalize the expression of miRNAs, we calculated the expression of five miRNAs relative to U6 expression. The specific primers used in this study are listed in Additional file 7: Table S1.

### Western blotting

Cells were collected by sample buffer and analyzed by electrophoresis. Proteins were transferred to polyvinylidene difluoride (PVDF, Merck Millipore) membrane and TBST buffer (10 mM Tris–HCl, pH 8.0, 150 mM NaCl, 0.05% Tween 20) containing 5% nonfat milk was used for blocking. Anti-Sp1 (Merck Millipore, 1:3000), anti-actin (Cat# 110,564, GeneTex, 1:5000), anti-GFP (Cat#sc-9996, Santa Cruz, 1:5000), anti-CD44 (GeneTex, 1:2000), anti-β-catenin (Cell Signaling, 1:1000), anti-ALDH1 (BD Biosciences, 1:1000), anti-SUMO-1 (ABclonal, Cambridge, MA, USA, 1:1000), anti-RNF4 (R&D systems, Minneapolis, MN, USA, 1:1000), anti-vimentin (Cell Signaling, 1:3000) and anti-E-cadherin (Cat#610,181, BD Biosciences, 1:1000) were used for probing interested proteins. After incubated with primary antibodies, PVDF membranes were then incubated with secondary immunoglobulin antibodies linked with horse radish peroxidase (Merck Millipore, 1:10,000). ECL Western blotting detection system (Merck Millipore) and ChemiDoc-it imager (UVP, Ultra-Violet Products Ltd., Cambridge, UK) were used for detecting signals.

### Transwell migration assay

The cell migration assay was performed using Transwell (Corning) system with an 8-μM pore size polycarbonate filter membrane. After overexpression of GFP, GFP-Sp1, or knockdown of Sp1 in A549 and E2-A549 cells for 24 h, cells were trypsinized and suspended in serum-free RPMI (Corning). Upper wells were filled with cell suspensions (2 × 10^4^) in serum-free RPMI (Corning) and lower wells were filled with RPMI (Corning) containing 10% FBS (Gibco). After incubation for 6 h at 37 °C, the lower side of filter membranes were fixed with 10% formaldehyde and stained with Giemsa (Merck Millipore). The migrated cells were counted under light microscope (Olympus).

### Wound healing assay

A549 cells were maintained in 6 cm dishes till 60% density and after overexpression of GFP and GFP-Sp1 by adenoviruses, adeno-GFP and adeno-GFP-Sp1, infection (MOI = 20) in cells for 48 h, or knockdown of Sp1 by lenti-shSp1 virus infection (MOI = 20) in cells for 72 h, the linear wound of cellular monolayer was created by scratching confluent cell monolayer using a plastic pipette tip. Cells were washed with PBS and photographed under microscopy observation. After incubation for another 24 h at 37 °C, migratory distance of cells was measures and relative migratory distance was analyzed [30].

### In vitro Matrigel-combined invasion assay

Cellular invasive property of cells was analyzed by invasion assay using the 24-well plate Transwell system with an 8 μM pore size polycarbonate filter membrane (Corning). The filter membrane was coated with 80 μl (1 mg/ml) of Matrigel (Cat#354,248, BD Biosciences). The cell suspensions were seeded to the upper compartment of the Transwell chamber at the cell density of 3 × 10^4^ in 100 μl of medium. After 24 h, the filter membrane was fixed with methanol for 10 min. The opposite surface of the filter membrane facing the lower chamber was stained with Giemsa (Merck Millipore) and the invasive cells were then counted under a microscope [31].

### Collection of specimens from lung cancer patients

After surgical resection at National Cheng Kung University Hospital, specimens of patients with lung adenocarcinomas were collected for immunohistochemical analysis or western blotting. The pathological data were analyzed by clinical pathologists.

### Protein stability assay

Cells were treated with 100 μg/ml cycloheximide (Sigma) to inhibit protein translation. Cells were resolved in sample buffer at indicated time, and protein stability was analyzed by western blotting. Protein level was quantified by using ImageJ software.

### Sphere formation assay

Standard sphere formation assays were performed according to Zhang et al. [32] with minor modification. The cells (1 × 10^3^) were resuspended in serum-free DMEM/F12 medium supplemented with 5 μg/ml insulin (Cat#I2643, Sigma), 20 ng/ml human recombinant epidermal growth factor (EGF; Cat#PHG0311, Gibco) and 10 ng/ml basic fibroblast growth factor (bFGF Cat#13,256–029, Gibco) in ultra-low attachment plates (Corning). Spheres that arose within 7–10 days were counted. Colony diameters > 50 μm were counted as a single-positive colony. The middle field was chosen for counting of spheres, and two fields for each plate were counted under a dissecting microscope.

### Luciferase reporter assay

1 × 10^5^ A549 or E2-A549 cells were seeded in each well of 6-well plates for 16 h, and adenoviruses, adeno-GFP and adeno-GFP-Sp1, were added as described. Two days after adenovirus infection, CD44P/pGL3 promoter-reporter construct (Addgene, Plasmid #19,122) or reporter plasmids containing aldehyde dehydrogenase 1 (ALDH1) and (sex determining region Y)-box 2 (Sox2) promoter regions were transfected into cells. Reporter plasmid DNA (1 μg) was co-transfected with Renilla Luciferase plasmid DNA (1 μg) (Promega, Mannheim, Germany) as a control for transfection efficiency. After 24 h incubation, transfected cells were lysed using the Dual-Luciferase Reporter Assay System (Promega). Promoter-driven luciferase activity was measured by luminometer (LB9506; Berthold Technologies, Bad Wildbad, Germany) and normalized to the Renilla Luciferase activity. Each experiment was carried out in triplicates and repeated three times. For construction, genomic DNA of A549 cells were prepared. The promoters of Sox2 and ALDH1 were produced by PCR using primers (Additional file 7: Table S1). After amplification and purification, the DNA fragments were ligated to pGL2 vector using restriction enzymes, KpnI/ NheI for pGL2-ALDH1 and pGL2-Sox2 (New England Biolabs, Ipswich, MA).

### DNA transfection

For transient transfection, the pcDNA3.0-GFP plasmids with the 3′UTRs of CD44s, CD44st, β-catenin and ALDH1, and pcDNA3.0-HA-tagged CD44s and CD44st were transfected into A549 and E2-A549 cells by PolyJet transfection reagent (Signagen. Laboratories, Ijamsville, MD, USA) and GFP-Sp1 overexpression by adenovirus, adeno-GFP-Sp1, infection (MOI = 20) for 48 h or knockdown of Sp1 by lenti-shSp1 virus infection for 72 h. For construction of pGL2-CD44s-3′UTR, CD44st-3′UTR, ALDH1-3′UTR, Sox2-3′UTR and β-catenin-3′UTR plasmids, the cDNA of A549 cells was prepared as the template for probing the 3′UTRs of CD44s, CD44st, ALDH1, Sox2 and β-catenin by PCR using primers (Additional file 7: Table S1). After amplification and purification, the DNA fragments were ligated to pGL2 vector using restriction enzymes, EcoRI/ BamHI.

### Chromatin immunoprecipitation coupled with sequencing (ChIP-Seq)

After crosslinking with 1% formaldehyde for 10 min at room temperature, A549 and E2-A549 cells (1 × 10^8^) were washed with ice-cold PBS three times and whole-cell extracts were prepared with lysis buffer. Samples were sonicated using a homogenizer (ultrasonic vibrator VCX 130; Sonics & Materials, Newtown, CT, USA); the condition was out put level of 4; 15 s on and 15 s off, total 3 min on ice at to shear chromatin to an average length between 300 to 500 bps. Samples were incubated with 20 μg of sonicated salmon sperm DNA for 2 h at 4℃ on a rotating device, then indicated antibodies (1:200) such as anti-ERβ (GTX70174, GeneTex, Irvine, CA, USA), anti-Sp1 (Cat#07–645, Merck Millipore) and anti-histone H3 (Cat#4620, Cell Signaling Technology, Danvers, MA, USA) antibodies were added and incubated for another 16 h on a rotating device. Beads were washed three times with high salt buffer (20 mM Tris HCl, 500 mM NaCl, 2 mM EDTA, 0.5% NP-40) and another three times with low salt buffer (10 mM Tris HCl, 100 mM NaCl, 1 mM EDTA, 0.5% NP-40, 0.01% SDS). Subsequently, proteins binding to beads were eluted by 500 μl of TE buffer containing 1% SDS, and crosslinks were reversed at 65 ℃ for 16 h. After protein digestion by 0.5 mg/ml proteinase K at 50 ℃ for 2 h, DNA was extracted by phenol/chloroform and precipitated by absolute alcohol at -20℃ for 30 min. The promoter of miR-3194 was detected by q-PCR. Primer sequences are listed in Additional file 7: Table S1. The DNA fragments recruited by anti-Sp1 antibody were sequenced (paired-end 35 bps) using the AB 5500xl SOLiD sequencer (Applied Biosystems, Carlsbad, CA, USA). Library construction and sequencing were performed by the Center for Bioinformatics and Digital Health at the National Cheng Kung University. The sequencing reads were trimmed and aligned to the genomic sequence retrieved from the reference human genome (hg19) using the CLC Genomics Workbench version 10.1.1 software (Qiagen, Hilden, Germany).

### Small RNA sequencing (small RNA-Seq)

Total RNA was extracted using TRIzol from scramble- or Sp1-knockdown A549 cells. The small RNA library preparation and sequencing (single-end 35 bps) using the AB 5500xl SOLiD sequencer were performed by the Center for Bioinformatics and Digital Health at the National Cheng Kung University. The raw reads were preprocessed by performing adapter trimming and then aligned to miRNA retrieved from miRbase (https://www.mirbase.org/) using the CLC Genomics Workbench version 10.1.1 software (Qiagen). The OncoMir database (http://www.oncomir.org/) was used to identify the targeting genes of miRNAs.

### Statistical analysis

All samples were used for statistical analysis. The difference between two groups was analyzed by Student’s t test; *p < 0.05, **p < 0.01, ***p < 0.005. Overall survivals were estimated by means of the Kaplan–Meier method and compared using the log-rank test. Univariate analyses were performed using the Cox risk proportion model. Hazard ratio (HR) with 95% confidence interval was used to study the survival rate. Statistical analyses were performed using the SPSS Statistics software V 17.0 (SPSS Inc., Chicago, IL, USA). The p-values, which was smaller than 0.05, were considered as statistically significant. Center value was defined as mean value, and S.E.M. was used to calculate and plot error bars from raw data. In addition, the Pearson Correlation analysis was used in this study; the r < 0 is negative correlation and r > 0 is positive correlation.

## Results

### Positive correlation between Sp1 level and survival rate in young women with lung cancer

**Fig. 1:**
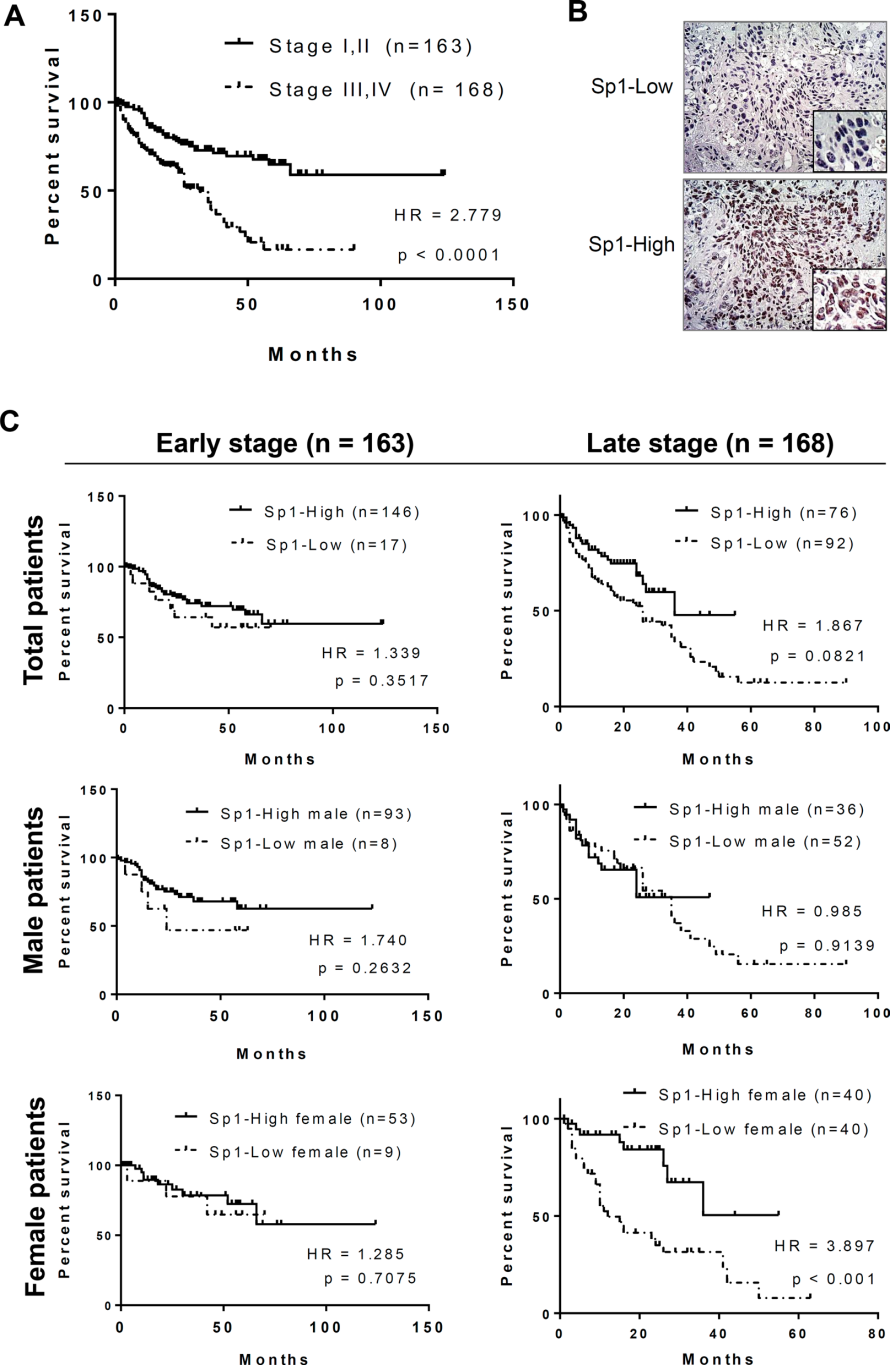
The Sp1 level is decreased in women with lung cancer and associated with a poor prognosis. The survival rates in the early- (stage I and II) and late-stage (stage III and IV) cohorts were analyzed by Kaplan–Meier survival curves and HR assay (**A**). The level of Sp1 in 331 lung cancer patients was studied by IHC, and then the samples were grouped into Sp1-Low and Sp1-High groups (**B**). The relevance between survival rate and Sp1 level in total patients (**C**, upper panel), male patients (**C**, middle panel) and female patients (**C**, lower panel) was analyzed by Kaplan–Meier survival curves and HR assay

**Fig. 2:**
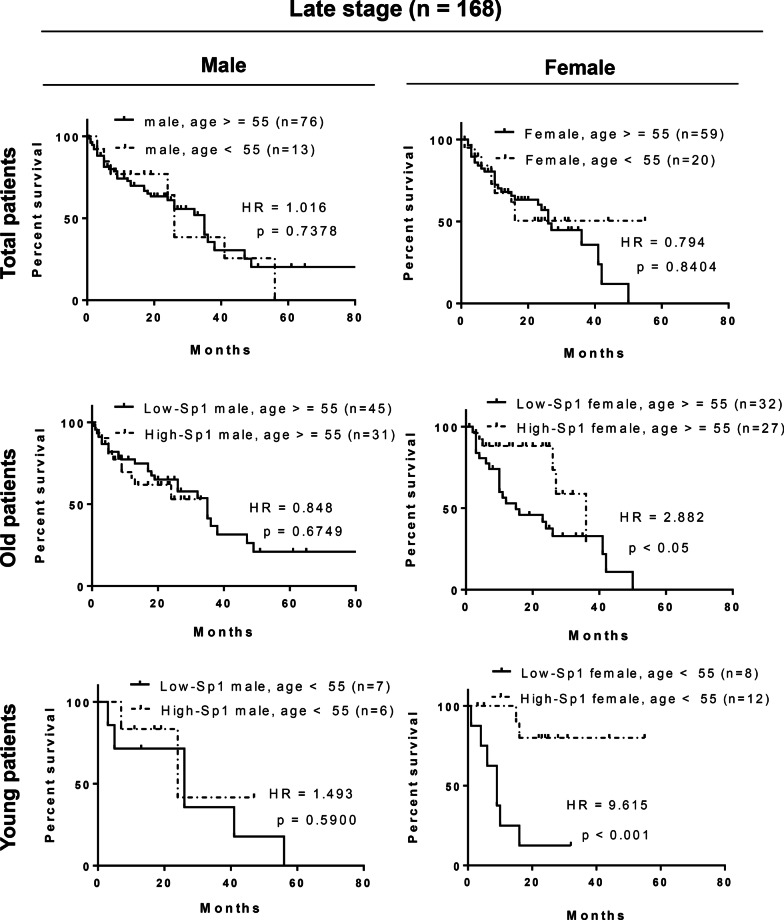
The Sp1 level is decreased in premenopausal women with lung cancer and associated with a poor prognosis. The survival rates of older (> = 55 years) and younger (< 55 years) male and female patients with late-stage lung cancer were analyzed by t test (upper panel). The survival rates of older (> = 55 years) (middle panel) and younger (< 55 years) (lower panel) patients with late-stage lung cancer and high and low Sp1 expression were analyzed by Kaplan–Meier survival curves and HR assays

**Fig. 3:**
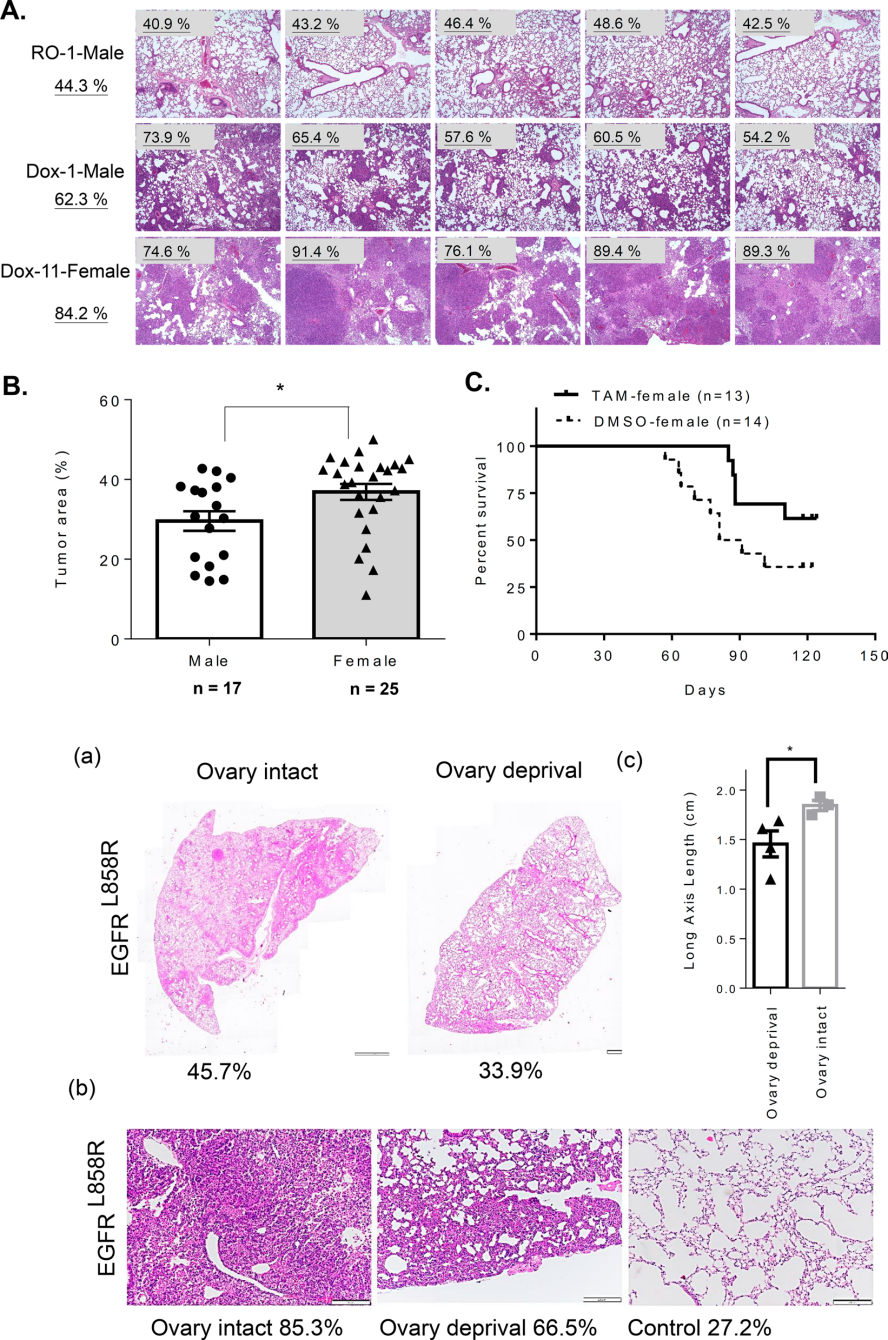
Sex dependence of lung cancer progression in a gender-dependent manner. Cancer formation was induced by water (RO-Male) or doxycycline (Dox-Male and Dox-Female) treatment for 1.5 months in mice with EGFR^L858R^-induced lung cancer mice. Cancer formation in the lung was studied by H&E staining, thus the lung area was quantitated by ImageJ software and listed on the left side (**A**). The tumor burden in 17 male mice (Dox-Male) and 25 female mice (Dox-Female) was quantitated by subtracting the lung area of normal mice (RO-mice), and statistically analyzed by a t test; *p < 0.05 (**B**). Female mice with EGFR^L858R^-induced lung cancer were treated with 10 mg/kg tamoxifen (TAM), intraperitoneal injection and one time every week to study the survival rate with Kaplan–Meier survival curves (**C**). Tumor formation in the lungs of EGFR^L858R^ mice (14 weeks of age) with or without ovary removal was studied by H&E staining including ×10 (**D**, (**a**)) and ×40 (**D**, (**b**)) images, and measurement of the axis length of lung size and statistically analyzed by a t test; *p < 0.05 (**D**, (**c**))

Our previous studies have indicated that the Sp1 levels are decreased in late-stage lung cancer and related to a poor prognosis [21]. In this study, we investigated the relationship among the Sp1 level, sex and survival rate of 331 lung cancer cohorts, including 185 male and 146 female cohorts by Kaplan–Meier survival curves and hazard ratio (HR) (Figs. 1, 2 and Additional file 7: Table S2). First, the prognosis was found to be poorer in patients with late-stage cancer than in those with early-stage cancer, and the HR was 2.779, consistent with previous studies (Fig. 1A). The level of Sp1 in all lung cancer cohorts was determined according to the Sp1 level in Fig. 1B. All cohorts were divided into early- and late-stage lung cancer groups and Sp1 level was studied by IHC assay. Based on the level of Sp1, the pathologists judged them as -, 1 + , 2 + and 3 + . Here we defined that lower Sp1 is—and 1 + , and higher Sp1 is 2 + and 3 + , subsequently divided them into two groups, higher Sp1 and lower Sp1. Sp1 was upregulated in most of the cohorts with early-stage lung cancer (89.6%), but not in those with late-stage lung cancer (45.2%) (Additional file 7: Table S2). The association between the survival rate and Sp1 level was studied (Fig. 1C). Sp1 downregulation in late-stage but not early-stage lung cancer patients was correlated with poor prognosis (HR = 1.867) (Fig. 1C, upper panel). When we studied the relationship between the Sp1 level and survival rate in a sex-dependent manner, we found that women with late-stage lung cancer and low Sp1 levels had a worse prognosis (HR = 3.897) than men with late-stage lung cancer and low Sp1 levels (HR = 0.985) (Fig. 1C, middle and lower panels), indicating that Sp1 is involved in lung cancer progression in a sex-dependent manner. Previous studies have also indicated that Sp1 can coregulate its target genes together with the ER [24, 33, 34]; therefore, in females with late-stage lung cancer, Sp1 may collaborate with the ER to regulate gene expression and thereby affect the survival rate. To study the relationship between the levels of Sp1 level and estrogen in women with lung cancer, patients with late-stage lung cancer were divided not only into male and female groups but also into older (> = 55 years) and younger (< 55 years) patient groups (Fig. 2). When prognosis was compared between the male and female patient groups, no difference in prognosis between the older and younger lung cancer patients was found (Fig. 2, upper panel). Next, in the older patient groups (> = 55 years), a low Sp1 level compared to a high Sp1 level was found to be related to a slightly poorer prognosis in women (HR = 2.882), but not in men (HR = 0.848), with late-stage lung cancer (Fig. 2, middle panel). Interestingly, in younger patients with late-stage lung cancer, a low Sp1 level was significantly associated with a poor prognosis in women (HR = 9.615) but not in men (HR = 1.493) with late-stage lung cancer, suggesting that premenopausal status, late-stage lung cancer, and low Sp1 expression are highly correlated with a poor prognosis (Fig. 2, lower panel). We also used EGFR^L858R^-induced lung cancer mice to address the role of estrogen in lung cancer progression (Fig. 3). First, we found that the tumor burden was higher in female mice (84.2%—44.3% = 39.9%) with EGFR^L858R^-induced lung cancer than in male mice (62.3%—44.3% = 18%) (Fig. 3A). After analyzing the tumor area of 17 male and 25 female mice, a higher tumor burden was found in female mice, indicating that estrogen may positively regulate lung cancer formation (Fig. 3B). The estrogen competitor tamoxifen was used to treat lung cancer formation in female mice with EGFR^L858R^-induced lung cancer (Fig. 3C). The data indicated that tamoxifen treatment increased the survival rate of female mice with lung cancer (p < 0.05), implying that estrogen might positively regulate lung cancer formation. To study the role of estrogen in lung cancer progression, the ovaries of EGFR^L858R^ female mice were removed by ovariectomy (Fig. 3D). The data indicated that loss of the ovaries decreased the tumor burden and long axis length of lung organs (Fig. 3D). Taken together, these data indicate that estrogen may positively regulate lung cancer progression and that Sp1 downregulation may be involved in this effect.

### Estrogen inhibits Sp1 to enhance lung cancer malignancy

**Fig. 4:**
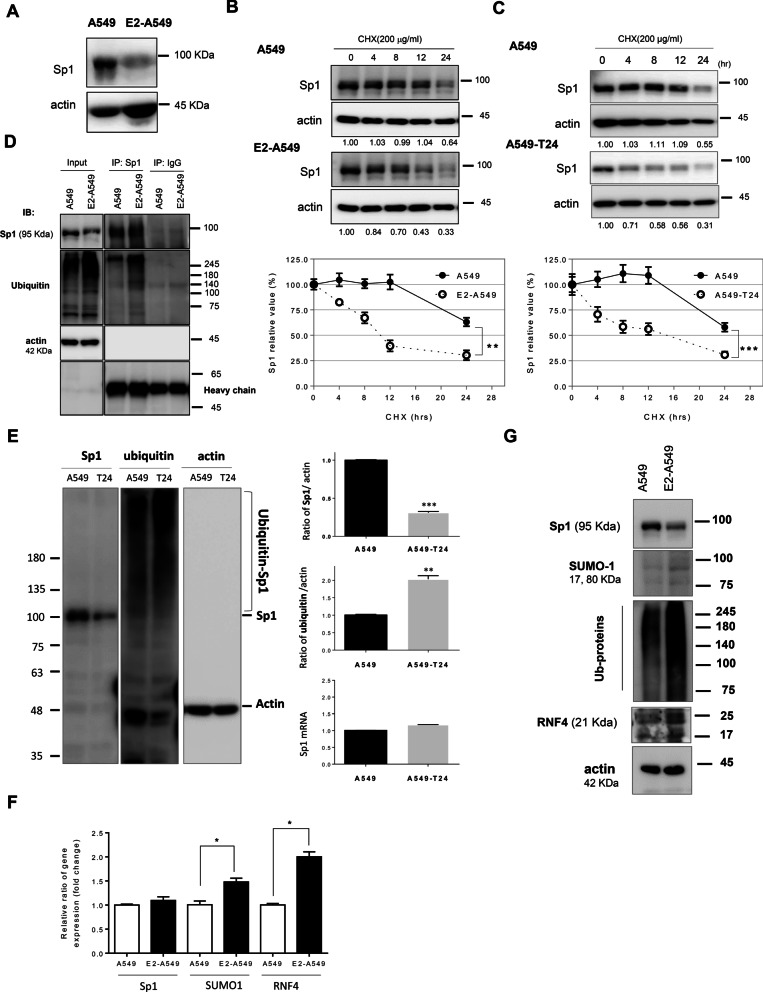
E2 decreases Sp1 protein stability in lung cancer cells. Samples of A549 and E2-A549 cells with or without cycloheximide treatment were used to study the indicated proteins by immunoblot analysis (**A**, **B**). Samples of A549 and A549-T24 cells with or without cycloheximide treatment were used to study the Sp1 level by immunoblot analysis (**C**). A549 cells were harvested to perform the immunoprecipitation (IP) experiments with anti-Sp1 antibodies; in addition, the ubiquitination of Sp1 was studied by Western blotting with anti-ubiquitin antibodies (**D**). Samples of A549 and A549-T24 cells were used to study the levels of Sp1 and ubiquitination signals by Western blotting with anti-Sp1 and anti-ubiquitin antibodies (**E**). Samples of A549 and E2-treated A549 cells were used to study the mRNA and protein levels of SUMO-1, RNF4 and ubiquitination signals by q-PCR and Western blotting with anti-Sp1, anti-SUMO-1, anti-RNF4 and anti-ubiquitin antibody (**F**, **G**). The results of three independent experiments were quantitated, and statistical analysis was performed with a t test; *p < 0.05, **p < 0.01, ***p < 0.005

Based on the results of Figs. 1, 2, 3, Sp1 and E2 may coregulate the progression of female lung cancer. However, what is the effect of E2 on Sp1 levels in lung cancer cells? The Sp1 level was significantly decreased in E2-A549 lung cancer cells (Fig. 4A). We then sought to elucidate the molecular mechanism underlying the decreased level of Sp1 in E2-A549 and A549-T24 lung cancer cells. Our previous studies found that RNF4 is the E3 ligase of Sp1 that regulates its stability [35]. Here, we found that the protein stability of Sp1 was decreased in E2-A549 cells (Fig. 4B) and A549-T24 cells (Fig. 4C). The ubiquitination signal of Sp1 was increased in E2-A549 (Fig. 4D) and A549-T24 lung cancer cells, but the mRNA level was unchanged in A549-T24 cells (Fig. 4E). Furthermore, we found that the mRNA and protein levels of RNF4 and small ubiquitin like modifier 1 (SUMO-1) were also increased in E2-A549 cells (Fig. 4F, G). Collectively, these results suggested that E2 increases the level of RNF4, thereby increasing the ubiquitination of Sp1 and resulting in its degradation in E2-A549 cells.

**Fig. 5:**
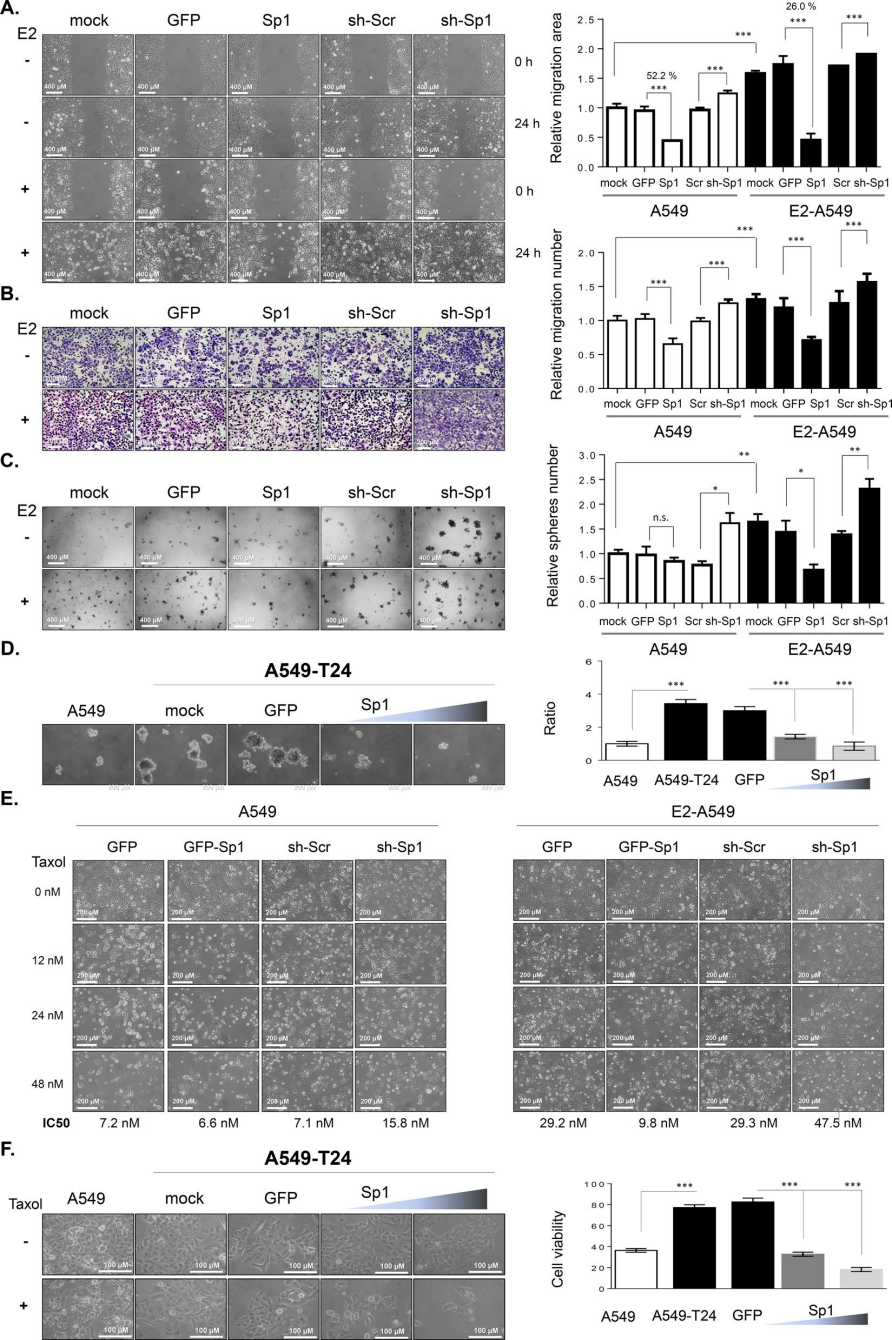
E2-mediated inhibition of Sp1 increases lung cancer cell progression. A549 and E2-A549 cells with or without Sp1 overexpression or knockdown were used to study migration activity by a wound healing assay (**A**) and chamber assay (**B**), and to study the cancer stemness by sphere formation assay (**C**). A549-T24 cells with or without Sp1 overexpression were used to study the cancer stemness by sphere formation (**D**). The cytotoxicity of Taxol in E2-A549 (**E**) and A549-T24 (**F**) cells with or without overexpression or knockdown of Sp1 was studied by a cell counting assay. The results of three independent experiments were quantitated, and statistical analysis was performed with a t test; *p < 0.05, **p < 0.01, ***p < 0.005

To investigate the role of Sp1 in E2-mediated lung cancer progression, we first studied the roles of E2 and Sp1 in lung cancer progression and drug resistance in vitro. Here, we found that E2 treatment significantly increased the migration ability of lung cancer cells (Fig. 5A). Overexpression of GFP-Sp1 decreased the migration ability of lung cancer cells, and knockdown of Sp1 increased the migration ability of A549 and E2-A549 lung cancer cells in wound healing (Fig. 5A and Additional file 2: Fig. S1) and chamber migration assays (Fig. 5B), suggesting that Sp1 negatively regulates the migratory ability of lung cancer cells. Because cancer stemness characteristics are the major reason for drug resistance and cancer recurrence, which lead to a poor prognosis, the role of Sp1 in sphere formation was studied in E2-A549 (Fig. 5C) and A549-T24 cells (Fig. 5D). The results indicated that overexpression of Sp1 decreased sphere formation and that knockdown of Sp1 increased sphere formation in E2-A549 cells (Fig. 5C). Overexpression of Sp1 also increased sphere formation in A549-T24 cells (Fig. 5D). Taken together, Sp1 decreases stemness characteristics and thus may block drug resistance and inhibit cancer malignancy. E2-A549 cells and A549-T24 cells were found to be resistant to Taxol cytotoxicity (Fig. 5E, F). Overexpression of GFP-Sp1 increased Taxol cytotoxicity, and Sp1 knockdown decreased Taxol cytotoxicity in A549 and E2-treated A549 cells (Fig. 5E). In addition, we also found that overexpression of GFP-Sp1 sensitized the drug-resistant lung cancer cell line A549-T24 to Taxol cytotoxicity (Fig. 5F). In summary, Sp1 negatively regulates lung cancer migration and stemness abilities in women with lung cancer, thus increasing Taxol-induced cytotoxicity.

### Sp1 inhibits CD44 expression in lung cancer cells

**Fig. 6:**
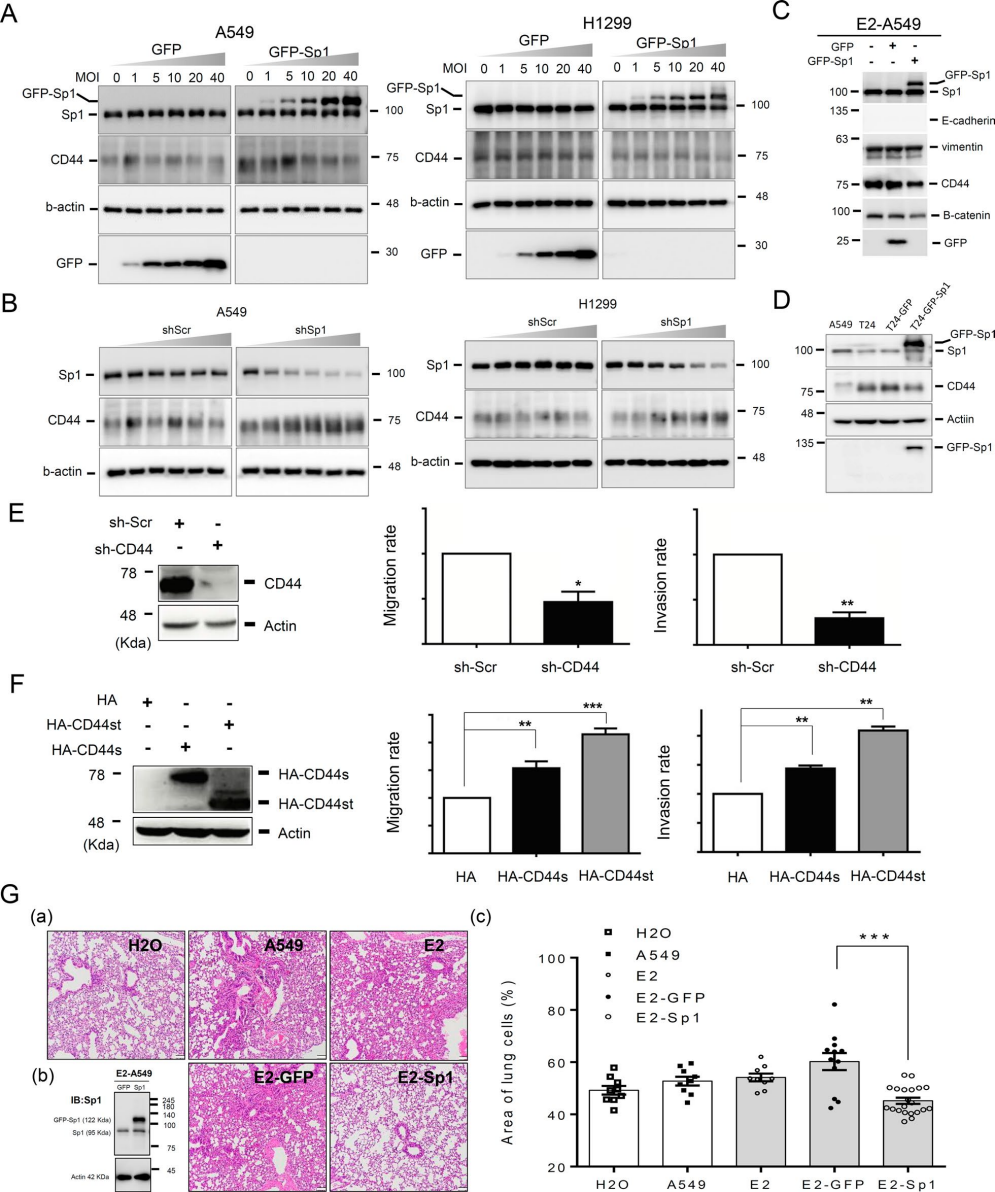
Sp1 inhibits CD44 expression in lung cancer. Samples of A549, H1299, E2-A549 and A549-T24 cells with or without Sp1 overexpression or knockdown were used to study the levels of Sp1 and CD44 by Western blotting with the indicated antibodies (**A**, **B**). Samples of A549, E2-A549 (**C**) and A549-T24 (**D**) cells were used to study the levels of E-cadherin, vimentin, CD44 and β-catenin by Western blotting with the indicated antibodies. The migration (**E**, **F**, middle panel) and invasion (**E**, **F**, right panel) abilities of A549 cells with or without CD44 knockdown (**E**) or overexpression (**F**) were studied by a chamber assay. GFP and GFP-Sp1 were overexpressed in A549 cells with or without E2 treatment and then injected into SCID mice via the tail vein for 5 weeks. Mice were sacrificed for H&E staining (**G**, **a**). The level of GFP-Sp1 was studied by Western blotting with an anti-Sp1 antibody (**G**, **b**). The tumor area was measured by ImageJ, and a statistical assay was performed (**G**, **c**). The results of three independent experiments were quantitated, and statistical analysis was performed with a t test; *p < 0.05, **p < 0.01, ***p < 0.005

**Fig. 7:**
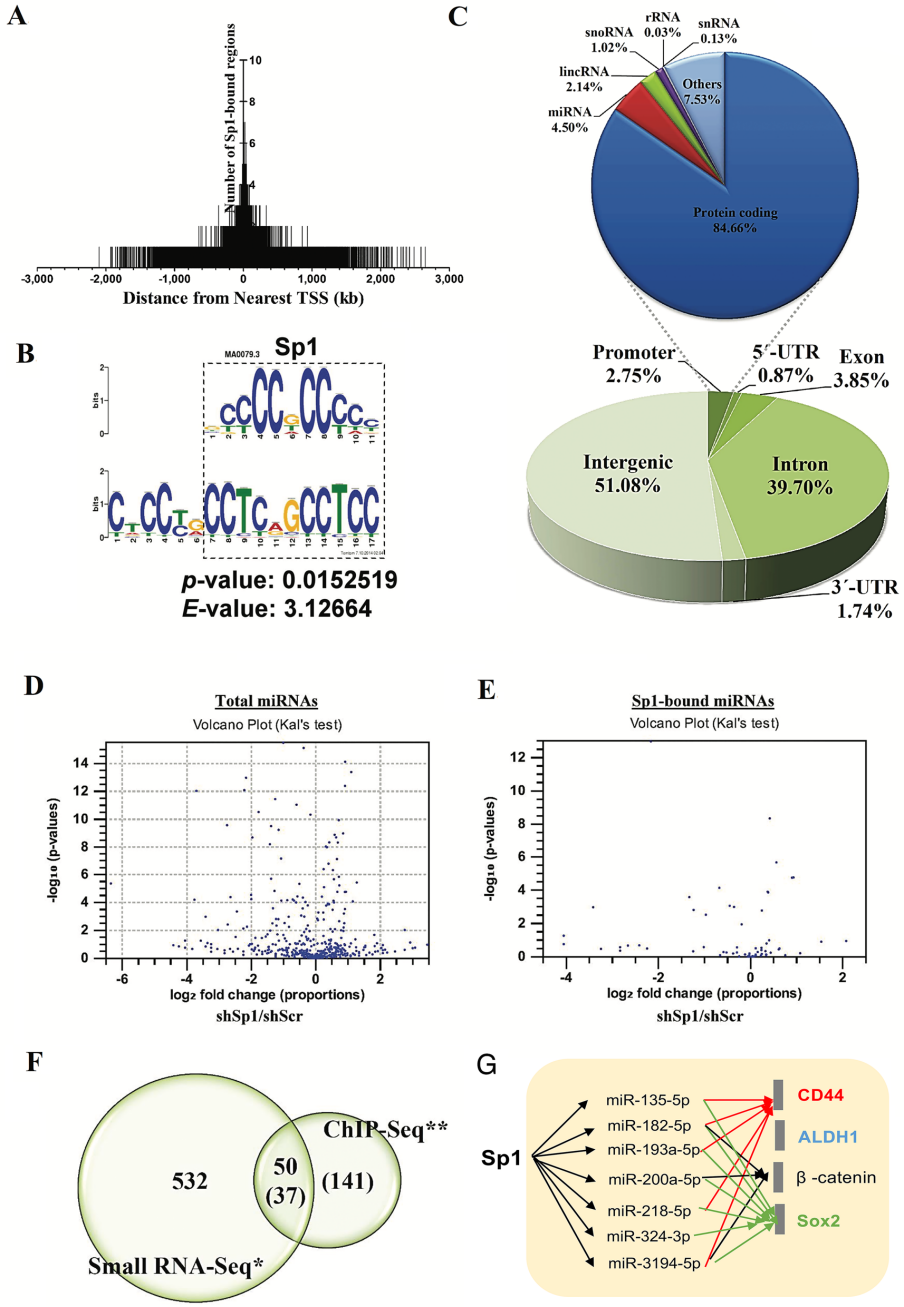
Global gene repertoire of miRNAs directly positively regulated by Sp1. The global repertoire of genes binding to Sp1 was studied by ChIP-Seq (**A**), and the binding sequence of Sp1 is shown here (**B**). The genomic region for Sp1 binding was analyzed (**C**). Genes positively regulated by Sp1 were studied by small RNA sequencing (**D**). The repertoire of miRNAs binding to Sp1 was studied by ChIP-Seq (**E**). The intersection between miRNAs positively and negatively regulated by Sp1 recruitment (**F**). Sp1-mediated miRNAs were analyzed by OncoMir to predict the candidate miRNAs targeting CD44, ALDH1, β-catenin and Sox2 (**G**)

Because CD44 is related to cancer stemness and cancer malignancy, the regulatory effect of Sp1 on the level of CD44 was studied (Fig. 6). Overexpression of GFP-Sp1 decreased the CD44 level (Fig. 6A), and Sp1 knockdown increased the CD44 level (Fig. 6B) in A549 and H1299 lung cancer cell lines, indicating that Sp1 negatively regulates CD44 expression in lung cancer cells. GFP-Sp1 overexpression also decreased the CD44 level in E2-A549 and A549-T24 cells but could not rescue the level of E-cadherin, which was inhibited by E2 treatment (Fig. 5C, D and Additional file 2: Fig. S2A). In addition, we also studied the level of E-cadherin in EGFR^L858R^-induced lung cancer mice. The data indicated that the level of E-cadherin in male mice was higher than that in female mice (Additional file 2: Fig. S2B). Knockdown of CD44 reduced the migration and invasion abilities of lung cancer cells (Fig. 6E); on the other hand, overexpression of HA-CD44, including the short form, HA-CD44st, and standard form, HA-CD44s, increased the migration and invasion abilities of lung cancer cells (Fig. 6F), indicating that Sp1-mediated inhibition of CD44 decreases the metastatic ability of lung cancer cells. To study the role of Sp1 in lung cancer progression in vivo, A549 cells with or without GFP-Sp1 overexpression and E2 treatment were injected into the tail vein of SCID mice to evaluate lung metastasis (Fig. 6G). The data indicated that E2 treatment increased the tumor area in the lung, but GFP-Sp1 overexpression reversed this effect, suggesting that Sp1 negatively regulates lung cancer metastasis. According to previous studies, Sp1 positively regulates the expression of most of its target genes directly; however, here, Sp1 negatively regulated CD44 expression. Therefore, we hypothesized that Sp1 might increase miRNA expression to silence CD44 expression. The miRNA expression levels and Sp1 binding repertoire were determined by small RNA sequencing (small RNA-Seq) and chromatin immunoprecipitation sequencing (ChIP-Seq), respectively (Fig. 7 and Additional file 7: Tables S3–S5). Most Sp1 binding motifs were localized within gene promoters (-2 kb to + 2 kb) (Fig. 7A), and 4.5% of Sp1 binding motifs were found within genomic loci for miRNA production (Fig. 7C). The typical binding motifs of Sp1 are 5′CCCCGCCCC3′ and 5′CCTCAGCCTCC3′ (Fig. 7B). Through small RNA-Seq analysis, we found that 532 genes containing miRNAs were regulated by Sp1 (Fig. 7D). By ChIP-Seq, we found that Sp1 was directly recruited to 141 genes for miRNA expression (Fig. 7E). Thirty-seven mRNAs specifically, 28 Sp1-positive and 9 Sp1-negative regulatory mRNAs, which were previously found to be involved in the regulation of various critical genes (Additional file 3: Fig. S3), were directly regulated by Sp1 (Fig. 7F and Table 1). Therefore, the OncoMir database (http://www.oncomir.org/) was used to identify candidate miRNAs targeting CD44, ALDH1, β-catenin and Sox2, including miR-3194-5p, miR-218-5p, miR-200-5p, miR-324-3p, miR-193a-5p, miR-182-5p and miR-135-5p (Fig. 7G).

**Table 1:** Positive regulation of miRNAs expression by Sp1

miRNA	Sequence	Fold change (shSp1/shScr)*	Sp1-bound position**
miR-324-3p	ACTGCCCCAGGTGCTGCTGG	− 16.61	− 595
miR-135-5p	TATGGCTTTTTATTCCTATGTGA	− 16.61	− 59
miR-223*	CGTGTATTTGACAAGCTGAGTT	− 10.68	− 1108
miR-3922-5p	TCAAGGCCAGAGGTCCCACAGCA	− 9.49	+ 5
miR-2116*	CCTCCCATGCCAAGAACTCCC	− 7.12	− 389
miR-187	TCGTGTCTTGTGTTGCAGCCGG	− 7.12	+ 179
miR-494-5p	AGGTTGTCCGTGTTGTCTTCTCT	− 6.33	− 322
miR-2116	GGTTCTTAGCATAGGAGGTCT	− 5.34	− 389
miR-4484	AAAAGGCGGGAGAAGCCCCA	− 4.75	− 1022
miR-22	AAGCTGCCAGTTGAAGAACTGT	− 4.71	− 741
miR-223	TGTCAGTTTGTCAAATACCCCA	− 4.61	− 1108
let-7i	TGAGGTAGTAGTTTGTGCTGTT	− 4.48	− 744
miR-21*	CAACACCAGTCGATGGGCTGT	− 2.61	− 363
miR-379	TGGTAGACTATGGAACGTAGG	− 2.51	− 531
miR-548Q-5p	GCTGGTGCAAAAGTAATGGCGG	− 2.37	− 1506
miR-629	TGGGTTTACGTTGGGAGAACT	− 2.36	− 1699
miR-129-3p	AAGCCCTTACCCCAAAAAGCAT	− 2.02	− 484
miR-1307	ACTCGGCGTGGCGTCGGTCGTG	− 1.96	+ 197
miR-22*	AGTTCTTCAGTGGCAAGCTTTA	− 1.82	− 741
miR-33b*	CAGTGCCTCGGCAGTGCAGCCC	− 1.60	− 1143
miR-3194	GGCCAGCCACCAGGAGGGCTG	− 1.58	− 1844
miR-4423-5p	AGTTGCCTTTTTGTTCCCATGC	− 1.50	− 1237
miR-4423-3p	ATAGGCACCAAAAAGCAACAA	− 1.44	− 1237
miR-218-5p	TTGTGCTTGATCTAACCATGT	− 1.37	− 1087
miR-3158	AAGGGCTTCCTCTCTGCAGGAC	− 1.30	− 1422
miR-182-5p	TTTGGCAATGGTAGAACTCACACT	− 1.28	− 1310
miR-182*	TGGTTCTAGACTTGCCAACTA	− 1.23	− 1310
miR-769-5p	TGAGACCTCTGGGTTCTGAGCT	− 1.23	− 924

The global levels of miRNAs in Sp1-overexpression A549 cells were determined by small RNA sequencing. The gene repertoire of Sp1 binding in A549 cells was determined by ChIP-Seq. The miRNA repertoire listed here includes miRNAs that can be directly positively regulated by Sp1

^*^The fold change between shSp1 and shScr normalized values from small RNA-seq data

^**^ The position of Sp1-bound regions with respect to the miRNA TSS

### Sp1 induces miRNA expression to silence CD44 expression

**Fig. 8:**
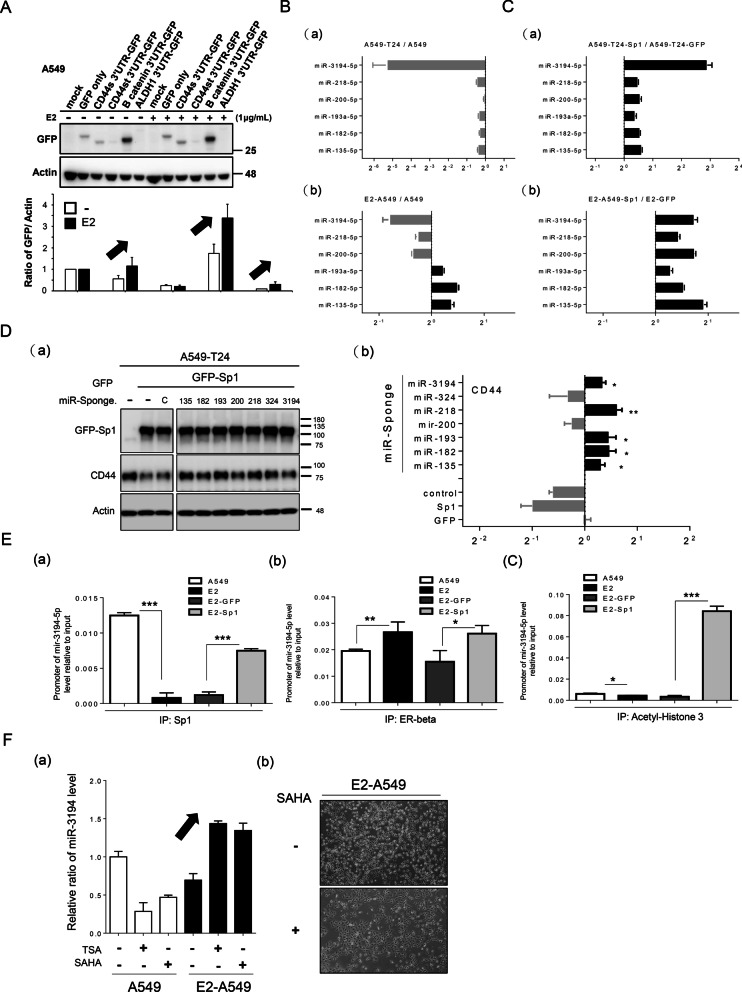
Sp1 positively regulates miRNAs to silence EMT-related gene expression. The 3′UTRs of various EMT-related genes, CD44s-3′UTR, CD44st-3′UTR, β-catenin-3′UTR and ALDH1-3′UTR, were fused to the 3′ end of GFP to study the GFP level in A549 and E2-A549 cells (**A**, **a**). After three independent experiments were finished, the levels of GFP were quantitated (**A**, **b**). The levels of various Sp1 regulated miRNAs, miR-3194-5p, miR-218-5p, miR-200-5p, miR-193a-5p, miR-182-5p and miR-135-5p, in A549, A549-T24 (**B**) and E2-A549 (**C**) cells with or without Sp1 overexpression were studied by q-PCR. After three independent experiments were completed, the levels of miRNAs were quantitated (**B**, **C**, **b**). The level of CD44 in A549 cells with or without overexpression of Sp1 and expression of the indicated miR sponges were studied by Western blotting (**D**, **a**). After three independent experiments were finished, the levels of CD44 were quantitated, and statistical analysis was performed with a t test; *p < 0.05 and **p < 0.01. The recruitment of Sp1 (**E**, **a**), ERβ (**E**, **b**) and acetyl histone 3 (**E**, **c**) to the promoter of miR-3194-5p was studied by ChIP assay. After three independent experiments were finished, the results were quantitated, and statistical analysis was performed with a t test; *p < 0.05, **p < 0.01, ***p < 0.005. The level of miR-3194-5p in A549 cells with or without E2, SAHA and TSA treatment was studied by q-PCR (**F**, **a**), and the morphology of E2-A549 cells with or without SAHA treatment was evaluated by ×20 microscopy (**F**, **b**). After three independent experiments were completed, the results were quantitated

Here, we clarify the mechanism by which Sp1 inhibits CD44. First, we found that the luciferase activity driven by the promoters of CD44, Sox2 and ALDH1 did not clearly change in E2-A549 (Additional file 4: Fig. S4A), GFP-Sp1-expressing and Sp1 knockdown A549 cancer cells (Additional file 4: Fig. S4B and C), implying that Sp1-mediated inhibition of EMT-related gene expression may occur through the 3′UTRs of CD44, β-catenin and ALDH1. The 3′UTRs of CD44, including CD44s, CD44st, β-catenin and ALDH1 were fused to GFP to construct pcDNA3.0-GFP-3′UTRs to individually study the effect of the 3′UTRs on Sp1-mediated inhibition of CD44, β-catenin and ALDH1 expression (Fig. 8A). We found that E2 did not affect the level of GFP level lacking a 3′UTR or GFP with the 3′UTR of CD44st but that the expression of GFP was increased with the 3´UTRs of CD44s, β-catenin and ALDH1, implying that E2 may decrease the expression of miRNAs that can silence the expression of CD44, β-catenin and ALDH1 (Fig. 8A). The levels of the Sp1-regulated candidate miRNAs targeting CD44, β-catenin and ALDH1, i.e., miR-3194-5p, miR-218-5p, miR-200-5p, miR-193a-5p, miR-182-5p and miR-135-5p, were studied in A549-T24 and E2-A549 lung cancer cells (Fig. 8B, C and Additional file 5: Fig. S5). The level of miR-3194-5p was significantly reduced in A549-T24 cells and E2-A549 cells (Fig. 8B(a) and C(a)); in addition, Sp1 positively regulated all the miRNAs, especially miR-3194-5p in E2-A549 and A549-T24 lung cancer cells (Fig. 8B(b) and C(b)). By using miRNA sponges to block the various Sp1-mediated miRNAs, we found that blockade of miR-3194-5p, miR-218-5p, miR-193-5p, miR-182-5p and miR-135-5p abolished the inhibitory effect of Sp1 on CD44 expression (Fig. 8D). E2 treatment decreased the recruitment of Sp1 and acetyl histone 3 but increased the recruitment of the ER to the promoter of miR-3194-5p (Fig. 8E). Overexpression of Sp1 increased the recruitment of Sp1 and acetyl histone 3 (Fig. 8E). E2 treatment in A549 cells decreased miR-3194-5p expression, but this effect was abolished by treatment with the HDAC inhibitors TSA and SAHA (Fig. 8F(a)). In addition, SAHA altered the morphology of E2-treated A549 cells, implying that ER may facilitate the recruitment of HDACs to the promoter of miR3194-5p to repress the expression of miR-3194-5p (Fig. 8F(b)). Taken together, these findings indicated that E2-mediated inhibition of Sp1 decreases miR-3194-5p expression to enhance CD44 expression.

**Fig. 9:**
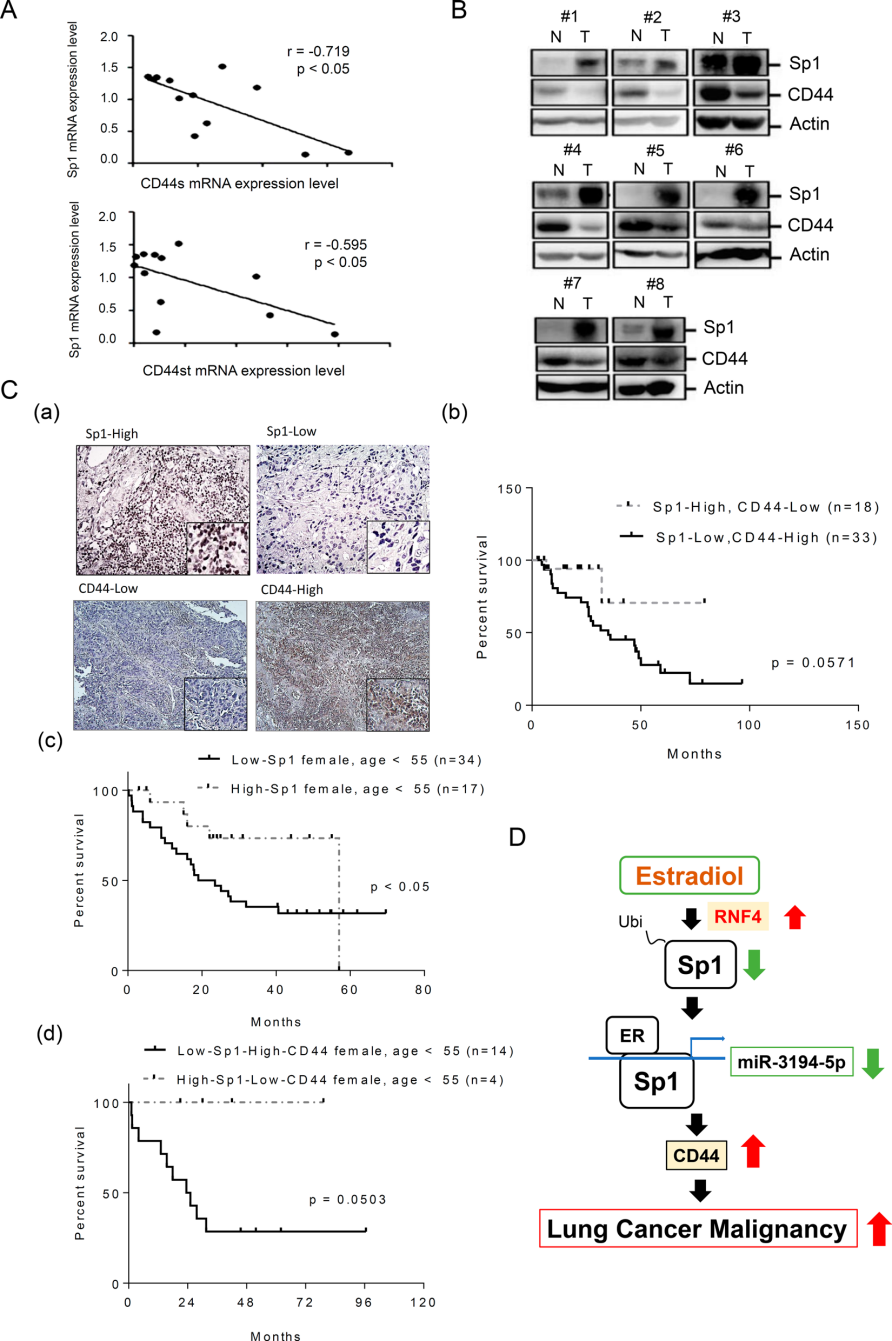
Clinical relevance. Samples obtained from lung cancer patients were used to study the mRNAs of Sp1 and CD44 including the standard form (CD44s) and short form (CD44st) by q-PCR. After three independent experiments were completed, the results were quantitated, and statistical analysis was performed with a Pearson Correlation analysis, r < 0: negative correlation (**A**). Samples of normal and tumor tissues obtained from 8 cohorts were used to study the levels of Sp1 and CD44 by Western blotting with anti-Sp1 and anti-CD44 antibodies (**B**). The levels of Sp1 and CD44 in 51 lung cancer clinical cohorts were studied by IHC. All the cohorts were divided into two groups, high and low, based on the signal (**C**, **a**), and the survival rates (**C**, **b**) of women and men with lung cancer based on Sp1 and CD44 levels were analyzed. The survival rates of younger (< 55 years) lung cancer patients with Sp1-low or Sp1-high (**C**, **c**), Sp1-low/CD44-high or Sp1-high/CD44-low (**C**, **d**) levels were analyzed with Kaplan–Meier survival curves. Working model: E2 increases RNF4 expression to enhance Sp1 degradation, thereby decreasing miR-3194-5p expression and resulting in an increase in CD44 expression in younger female lung cancer patients (**D**)

The relationship between Sp1 and CD44 levels was studied in normal and tumor clinical tissues. The mRNA levels of Sp1 and CD44, including CD44s and CD44st, exhibited inverse correlations in the clinical lung cancer cohorts (Fig. 9A). Eight lung cancer specimens collected from lung cancer patients were used to study the protein levels of Sp1 and CD44. An inverse correlation between Sp1 and CD44 protein levels was found in 7 of 8 specimens (Fig. 9B). The protein levels of Sp1 and EMT-related markers, including CD44, β-catenin and ALDH1, in 51 lung cancer patients were determined by IHC and grouped them into high and low expression individually, subsequently the relationship between the survival rate and protein levels was studied (Fig. 9C and Additional file 6: Fig. S6). The data indicated that patients with the Sp1-low/CD44-high phenotype had a significantly poorer prognosis than patients with the Sp1-high/CD44-low phenotype (Fig. 9C(a) and (b)), but there was no significant difference among patients with the Sp1-high/β-catenin-low, Sp1-low/β-catenin-high, Sp1-high/ALDH1-low and Sp1-low/ALDH1-high phenotypes (Additional file 6: Fig. S6). Next, we collected data on premenopausal women less than 55 years old with lung cancer to study the levels of Sp1 and CD44 (Fig. 9C(c) and (d)). The results indicated that young female lung cancer patients with the Sp1-low phenotype had a poorer prognosis than patients with the Sp1-high phenotype (Fig. 9C(c)). The data indicated that young female lung cancer patients with the Sp1-low/CD44-high phenotype had a significantly poorer prognosis than patients with the Sp1-high/CD44-low phenotype (Fig. 9C(d)).

## Discussion

The poor prognosis of female patients with lung cancer has recently become an important clinical issue [10]. In this study, we found that E2 increased RNF4 to reduce the Sp1 level, thereby enhancing CD44 expression through downregulation of miRNAs, leading to a poor prognosis in young women with lung cancer (Fig. 9D); this finding may be beneficial for developing therapeutic strategies in the future.

Previous studies have indicated that the expression of aromatase, a P450 enzyme that mediates the final and rate-limiting step in E2 synthesis to catalyze the conversion of testosterone to E2 [36], is increased in lung cancer patients. Aromatase overexpression is associated with poor prognosis and poor survival in both male and female patients with lung cancer [37]. High ERβ and aromatase levels are associated with poor clinical outcomes in NSCLC patients [38, 39]. Regarding the impact of the E2 receptor on NSCLC prognosis, ERα and ERβ have been identified in lung cancer patients. ERβ is the major type of ER in NSCLC. According to previous studies on the roles of E2 in physiological and pathological conditions, one kind of role is ER-dependent and the other involves ER-independent pathways that regulate cancer progression [40, 41]. Previous studies have revealed that E2 treatment induces rapid activation of the EGFR pathway, implying that nonnuclear ER regulates the EGFR pathway to influence lung cancer progression. Moreover, 67% of EGFR mutation positive tumors have high nuclear ERβ expression (versus 37% in EGFR wild-type tumors) [42]. Based on these previous studies, ER-mediated and EGFR-mediated signaling pathways may positively coregulate cancer-related gene expression during lung cancer progression. In this study, we first clarified the molecular mechanisms by which E2 is related to a poor prognosis in women with lung cancer, especially premenopausal women. Our study also indicated that E2 reduced Sp1 levels to inhibit miRNA expression, in turn enhancing CD44 expression, subsequently resulting in a poor prognosis in women with lung cancer. Therefore, both ER-dependent and ER-independent pathways may be regulated by E2 in parallel to regulate lung cancer progression. In addition, although we found that both E2-treated A549, E2-A549, and Taxol-induced drug resistant A549, A549-T24, could regulate EMT, but the regulatory mechanisms between them are distinguishable. First, both of them induced CD44 expression, but CD44 cleavage was only found in A549-T24 cells (Fig. 6D). Here, we also found that E2 regulates only the CD44s 3′UTR but not the CD44st 3′UTR. When we analyzed the possible miRNAs targeting the 3′UTRs of CD44s and CD44st, many different repertoires of miRNAs between the CD44s 3′UTR and CD44st 3′UTR were found, suggesting that the regulation of CD44s and CD44st is very different. In addition, the repertoire of Sp1-mediated miRNAs in E2-A549 and A549-T24 cells was distinct, implying that different regulatory mechanisms exist between these cells in regulating EMT. Finally, E-cadherin expression was nearly abolished in both of E2-A549 and A549-T24 cells but could not be rescued by Sp1, implying that Sp1 can regulate CD44, β-catenin and ALDH1 but cannot regulate E-cadherin. The regulatory mechanisms of how E2 treatment and Taxol treatment inhibit E-cadherin are interesting and need to be elucidated in the future.

Sp1 is an important transcription factor that regulates the expression of many genes involved in various physiological and pathological conditions, such as cancer progression. The expression level and DNA binding activity of Sp1 contribute to its transcriptional activity [43]. According to previous studies, complex posttranslational modifications of Sp1 such as phosphorylation, ubiquitination, acetylation and sumoylation can regulate its protein degradation, transactivity and DNA binding affinity, thus regulating the expression of its target genes [20]. Phosphorylation of Sp1 at Ser278 and Ser739 increases its protein stability but decreases its DNA binding affinity [27]. Sumoylation of Sp1 at Lys16 decreases the protein stability of Sp1 [28]. In addition, acetylation of Sp1 at Lys710 increases the recruitment of HDAC1/2 to the target to silence target gene expression [44]. Although Sp1 positively regulates cancer proliferation, our previous studies indicate that Sp1 negatively regulates lung cancer metastasis in the late stage [21]. In this study, we first found that there was an inverse correlation between the Sp1 level and lung cancer prognosis in young women but not in men or menopausal women. Although Sp1-low older female lung cancer patients also had a slightly poor prognosis (Fig. 2, middle panel), we speculate that this might be due to menopausal age. Here, we defined the menopausal age as 55 years old, which may be associated with some level of estrogen exposure. We had regrouped the late-stage cohorts based on 60 years old and then studied the relevance between the survival rate and Sp1 level. The data indicated that there was no difference in the survival rate of the older female patients between the Sp1-low and Sp1-high cohorts (data not shown). Interestingly, we found that E2 treatment could decrease Sp1 levels in lung cancer cells through a decrease in Sp1 protein stability. E2 treatment in A549 cells increased the expression of RNF4, which is the E3-ligase of Sp1 [35], possibly through E2-induced transcriptional activity of RNF4. RNF4 is a SUMO-targeted ubiquitin ligase that can be recruited to its substrates through the interaction between the SUMO motif of RNF4 and the sumoylation moiety on Sp1 [28, 35]. In addition, although we found that estrogen can increase RNF4 expression to increase Sp1 degradation, thereby decreasing miR-3194-5p expression, it is unclear whether estrogen can directly regulate miR-3194-5p expression. As shown in Fig. 8E, E2 nearly abolished the recruitment of Sp1 to the promoter of miR-3194-5p, possibly not only due to the decrease in Sp1 level but also due to the inhibition of the binding affinity of Sp1 to the promoter of miR-3194-5p. Sp1 has been reported to regulate the expression of not only many coding genes but also noncoding genes [45, 46]. Our previous studies have indicated that Sp1-mediated miR-182 expression silences FOXO3 to enhance lung cancer malignancy [19]. An increasing number of studies have revealed that Sp1 regulates the expression of many noncoding genes. Recent studies have indicated that Sp1-induced overexpression of LINC00520 facilitates NSCLC progression through the miR-577/CCNE2 pathway and predicts poor prognosis [47]. Sp1-induced AFAP1-AS1 contributes to proliferation and invasion by regulating the miR-497-5p/CELF1 pathway in nasopharyngeal carcinoma [48]. Another study also revealed that miR-4310 induced by Sp1 targets PTEN to promote glioma progression [49]. Understanding the detailed molecular mechanisms underlying the poor prognosis of women with lung cancer will be beneficial for the development of cancer therapy for these individuals in the future.

## Conclusions

The poor prognosis of female patients with lung cancer has recently become an important clinical issue. Herein we found that there was an inverse correlation between the Sp1 level and poor prognosis in the late-stage premenopausal female lung cancer patients. Investigation of the molecular mechanism showed that E2 increases RNF4 expression to reduce Sp1 levels, thereby enhancing CD44 expression through downregulation of several miRNAs, and this leads to a poor prognosis in young women with lung cancer. These findings may be beneficial for developing the therapeutic strategies in the future.

## Supplementary Information

**Additional file 1: Fig. S1.** The level of Sp1 in A549 and E2-A549 cells with or without Sp1 overexpression or knockdown was studied by Western blotting with anti-Sp1 and GFP antibodies.

**Additional file 2: Fig. S2.** The mRNA level of E-cadherin in A549 cells with or without E2 treatment was studied by q-PCR. After three independent experiments were completed, the results were quantitated, and statistical analysis was performed with a t test; *p < 0.05 (A). The level of E-cadherin in EGFR^L858R^-induced lung cancer mice including male and female mice was studied by IHC assay (B)

**Additional file 3: Fig. S3.** Sp1-regulated miRNAs are related to the indicated important proteins.

**Additional file 4: Fig. S4.** The promoter activities of CD44, Sox2 and ALDH1 were studied by luciferase assays in A549 and E2-A549 cells (A) and in E2-A549 cells with or without Sp1 overexpression (B) or knockdown (C).

**Additional file 5: Fig. S5.** The levels of the EMT-related markers CD44, Sox2, β-catenin and ALDH1 in A549-T24 cells with or without GFP-Sp1 overexpression and treatment with various miR sponges were studied by Western blot assay, and the results were quantitated after three independent experiments (A). Target genes of various indicated miRNAs (B).

**Additional file 6: Fig. S6.** The relationship between the survival rate and the levels of various proteins, including Sp1-low/β-catenin-high or Sp1-high/β-catenin-low levels (A) and Sp1-low/ALDH1-high or Sp1-high/ALDH1-low levels (B), in lung cancer cohorts was analyzed by Kaplan–Meier survival curves.

**Additional file 7:** Supplementary Tables.

## Data Availability

The datasets generated and/or analyzed during the current study are available from the corresponding author on reasonable request.

## Abbreviations

ALDH1: Aldehyde dehydrogenase
ChIP: Chromatin immunoprecipitation
E2: Estradiol
ECL: Enhanced chemiluminescence
EGFR: Epidermal growth factor receptor
EMT: Epithelial mesenchymal transition
ER: Estrogen receptor
FBS: Fetal bovine serum
GAPDH: Glyceraldehyde 3-phosphate dehydrogenase
GFP: Green fluorescent protein
HDAC: Histone deacetylase
HE staining: Hematoxylin–eosin staining
HR: Hazard ratio
IHC: Immunohistochemistry
miRNA: MicroRNA
NSCLC: Non-small-cell lung cancer
q-PCR: Quantitative polymerase chain reaction
RNF4: RING finger protein 4
SAHA: Suberoylanilide hydroxamic acid
SCID: Severe combined immunodeficiency
SCLC: Small cell lung cancer
S.E.M.: Standard error of the mean
Sox2: (Sex determining region Y)-box 2
SUMO-1: Small ubiquitin like modifier 1
TSA: Trichostatin A
