# Supplementary figures and images for: Estradiol-mediated inhibition of Sp1 decreases miR-3194-5p expression to enhance CD44 expression during lung cancer progression
Source: J Biomed Sci. 2022 Jan 17;29:3. doi: 10.1186/s12929-022-00787-1 (PMC8762881; doi:10.1186/s12929-022-00787-1)

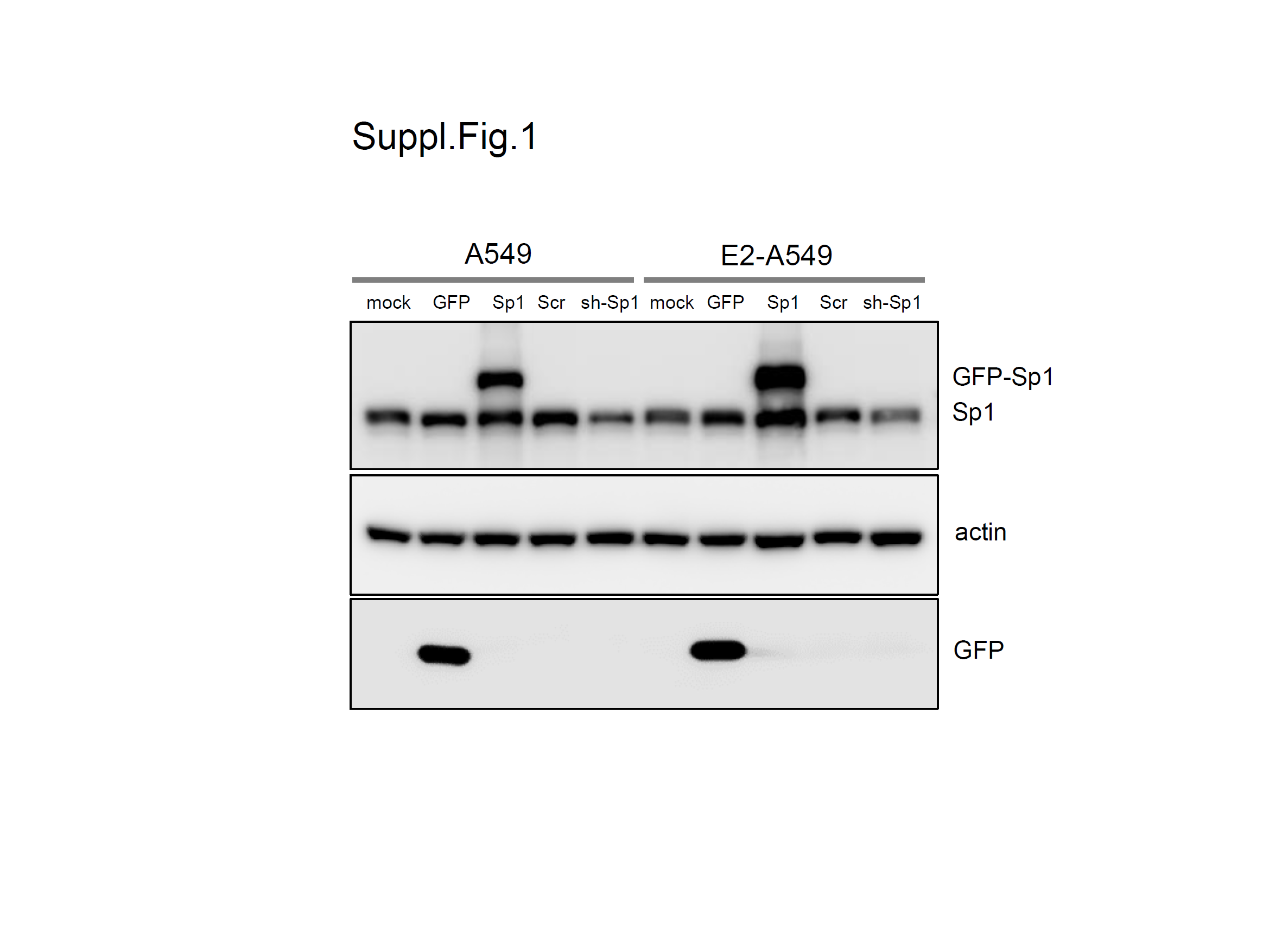

Supplement: Supplementary file 1 — Additional file 1: Fig. S1. The level of Sp1 in A549 and E2-A549 cells with or without Sp1 overexpression or knockdown was studied by Western blotting with anti-Sp1 and GFP antibodies. [file 12929_2022_787_MOESM1_ESM.tif]

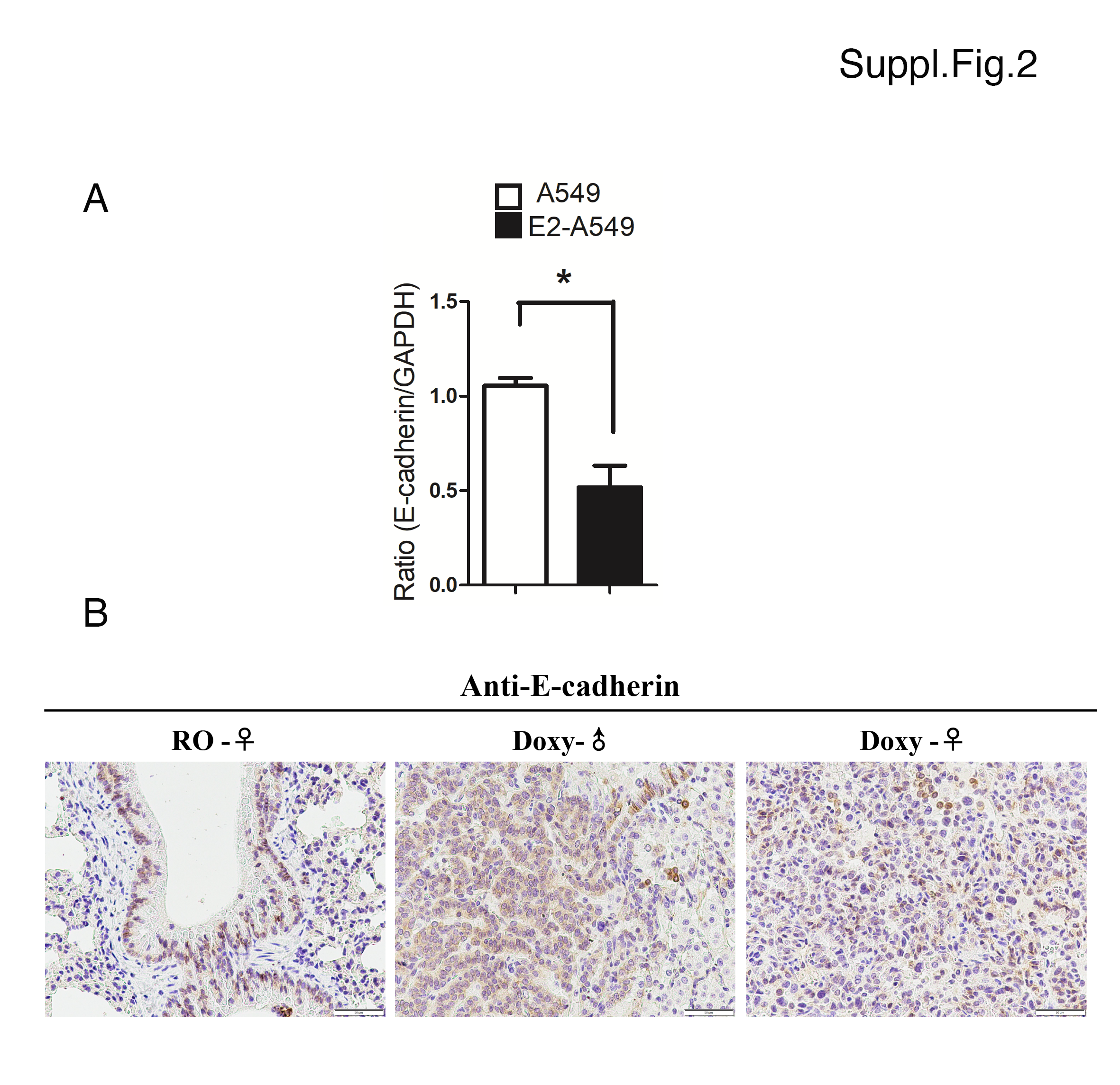

Supplement: Supplementary file 2 — Additional file 2: Fig. S2. The mRNA level of E-cadherin in A549 cells with or without E2 treatment was studied by q-PCR. After three independent experiments were completed, the results were quantitated, and statistical analysis was performed with a t test; *p < 0.05 (A). The level of E-cadherin in EGFRL858R-induced lung cancer mice including male and female mice was studied by IHC assay (B) [file 12929_2022_787_MOESM2_ESM.tif]

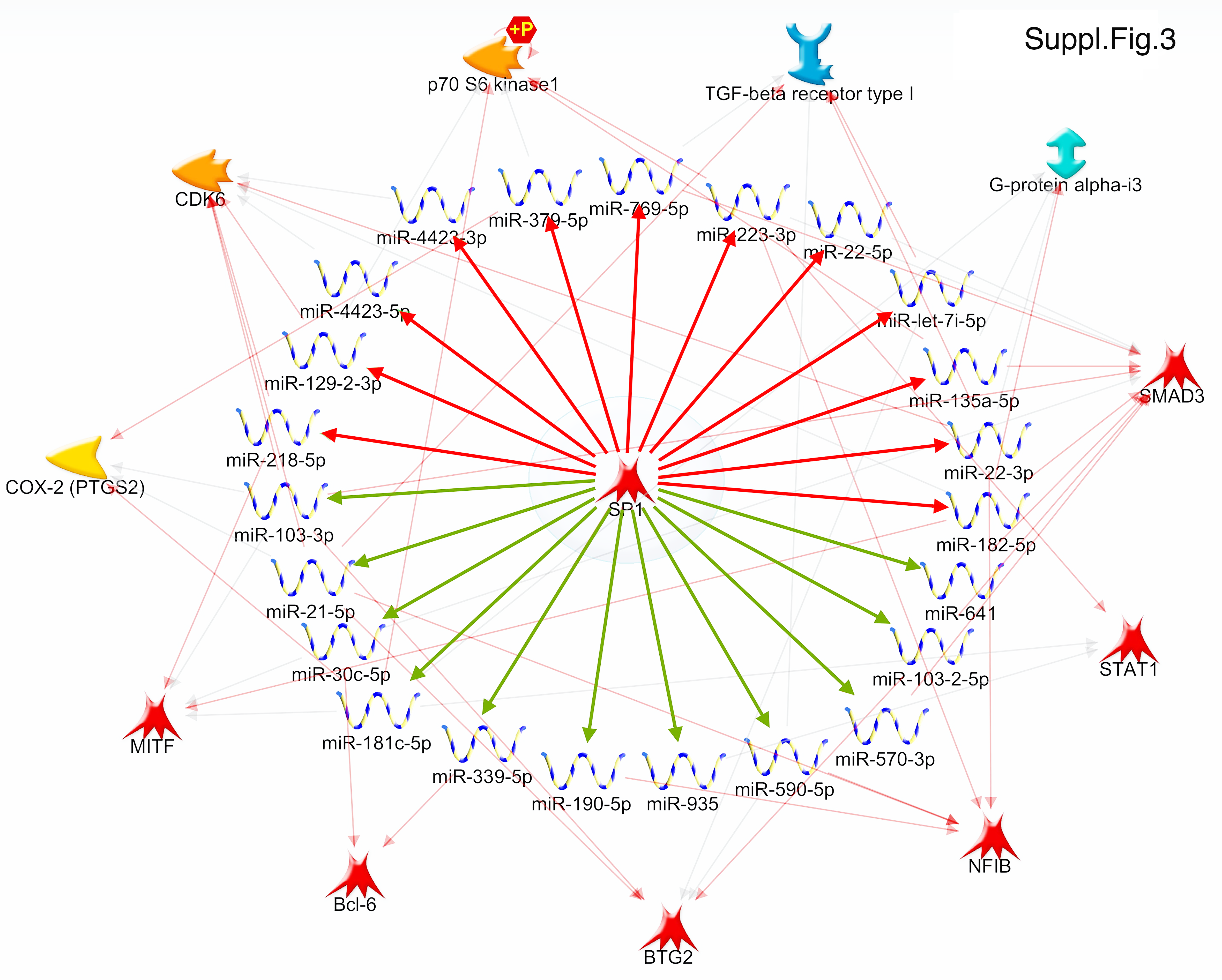

Supplement: Supplementary file 3 — Additional file 3: Fig. S3. Sp1-regulated miRNAs are related to the indicated important proteins. [file 12929_2022_787_MOESM3_ESM.tif]

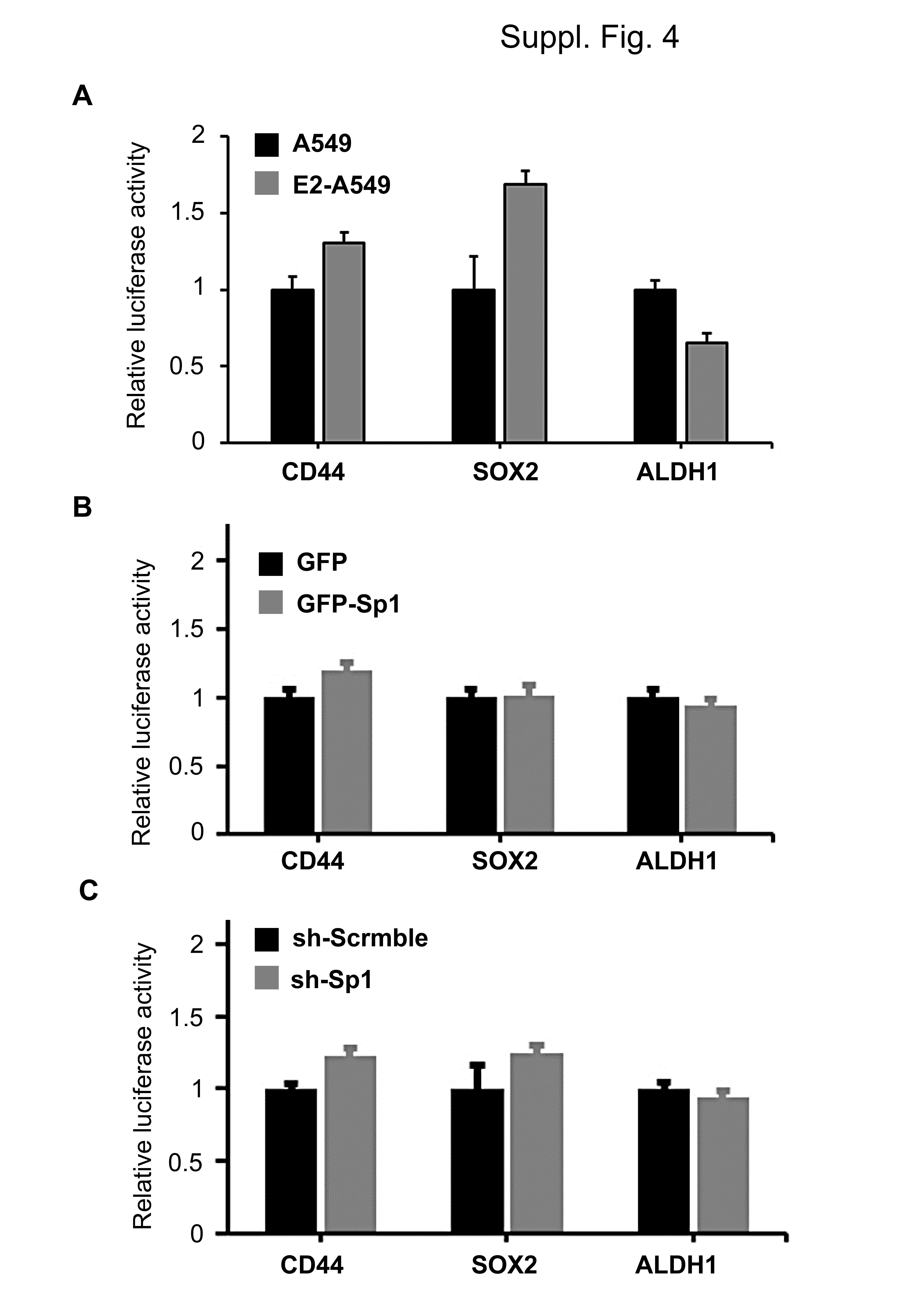

Supplement: Supplementary file 4 — Additional file 4: Fig. S4. The promoter activities of CD44, Sox2 and ALDH1 were studied by luciferase assays in A549 and E2-A549 cells (A) and in E2-A549 cells with or without Sp1 overexpression (B) or knockdown (C). [file 12929_2022_787_MOESM4_ESM.tif]
