# Supplementary material for: Estradiol-mediated inhibition of Sp1 decreases miR-3194-5p expression to enhance CD44 expression during lung cancer progression
Source: J Biomed Sci. 2022 Jan 17;29:3. doi: 10.1186/s12929-022-00787-1 (PMC8762881; doi:10.1186/s12929-022-00787-1)
Supplement: Supplementary file 7 — Additional file 7: Supplementary Tables. [file 12929_2022_787_MOESM7_ESM.doc]

**Supplementary Tables**

**Estradiol-mediated inhibition of Sp1 decreases miR-3194-5p expression to enhance CD44 expression during lung cancer progression**

**Young et al.**

**Supplementary Table 1.**

**Sequences of primers used for Q-PCR, Chip PCR and target sequence of shRNA-Sp1**

| **RT-PCR and Q-PCR primers** | | | |
| --- | --- | --- | --- |
| **Gene** | Forward primer | Reverse primer | |
| **Sp1** | 5′-GGCTCGGGGGATCCTGGC-3′ | 5′-TATGGCCCATATGTCTCTG-3′ | |
| **SUMO-1** | 5′-AGCAGTGAGATTCACTTCAAAGTG-3′ | 5′-TCTGACCCTCAAAGAGAAACCTG-3′ | |
| **RNF4** | 5′-GTCAGTTGTCCCATCTGCATGG-3′ | 5′-AATCACGGAGGCACTGGCTACA-3′ | |
| **GAPDH** | 5’-CCATCACCATCTTCCAGGAG-3’ | 5’-CCTGCTTCACCACCTTCTTG-3 | |
| **Human miR-3194-5p** | 5’-GGCCAGCCACCAGGAGGGCTG-3’ | mRQ 3’-primer | |
| **Human miR-218-5p** | 5’-TTGTGCTTGATCTAACCATGT-3’ | mRQ 3’-primer | |
| **Human miR-200-5p** | 5’-CATCTTACCGGACAGTGCTGGA-3’ | mRQ 3’-primer | |
| **Human miR-193a-5p** | 5’-TGGGTCTTTGCGGGCGAGATGA-3’ | mRQ 3’-primer | |
| **Human miR-182-5p** | 5’-TTTGGCAATGGTAGAACTCACACT-3’ | mRQ 3’-primer | |
| **Human miR-135-5p** | 5’-TATGGCTTTTTATTCCTATGTGA-3’ | mRQ 3’-primer | |
| **ChIP PCR primers** | | | |
| **miR-3194-5p promoter** | 5’-GGTACCAGTCCAAAGTTGTGTTTAC-3’ | 5’-CCTGAAGACACGCTGAGTA-3’ | |
| **shRNA-Sp1** | Target sequence | | |
|  | 5’-CCGGGCTGGTGGTGATGGAATACATCTCGAGATGTATTCCATCACCACCAGCTTTTTG-3 | | |
| **Promoter** | | | |
| **ALDH1-Forward** | 5’-GGTACCCTTGGTCCCTAGGTTCTCA-3’ | | |
| **ALDH1-Reverse** | 5’-GCTAGCCAGCTGCTCTGGCCACTAA-3’ | | |
| **Sox2-Forward** | 5’-GGTACCAAGAACTAAAACAAGCCAT-3’ | | |
| **Sox2-Reverse** | 5’-GCTAGCCATGTGACGGGGGC-3’ | | |
| **3’UTR** | | | |
| **CD44s 3’UTR-Forward** | 5’-GAATTCTCACCTACACCATTATCTT-3’ | | NM_000610.4 (2356 - 5413) |
| **CD44s 3’UTR-Reverse** | 5’-GGTACCCTGTTTCCTTTAGTCTTT-3’ | |  |
| **CD44st 3’UTR-Forward** | 5’-GAATTCTAGAGATTCAGGTTATAGC-3’ | | NM_001202557.2 (1021 - 1943) |
| **CD44st 3’UTR-Reverse** | 5’-GGTACCAGGAAATTCATATTTAAT-3’ | |  |
| **ALDH1 3’UTR-Forward** | 5’-GAATTCAGAAAATATAAGAGTGGAG-3’ | | NM_000689.5 (1560 - 2082) |
| **ALDH1 3’UTR-Reverse** | 5’-GGATCCTCATGCAAGGTTGTT-3’ | |  |
| **Sox2 3’UTR-Forward** | 5’-GAATTCGGGCCGGATAGCGAACTGG-3’ | | NM_003106.4 (1391 - 2498) |
| **Sox2 3’UTR-Reverse** | 5’-GGATCCTTTCAGTGTCCATAT-3’ | |  |
| **β-catenin 3’UTR-Forward** | 5’-GAATTCATCATCCTTAAGCTGTATT-3’ | | NM_001098209.2 (2568 - 3347) |
| **β-catenin 3’UTR-Reverse** | 5’-GGATCCCAATCGAATGAATTA-3’ | |  |

**Supplementary Table 2. Patients’ history**

| **Characteristic** | **High Sp1 expression of patients (n)** | **Low Sp1 expression of patients (n)** | **P-value** |
| --- | --- | --- | --- |
| Age  (mean ± s.e.m.) | 64.9 ± 0.9 | 64.6 ± 1.4 | 0.417 ^a^ |
| Male | 131 (70.8%) | 54(29.2%) | 0.130 ^b^ |
| Female | 91 (62.3%) | 55(37.7%) |  |
| Tumor stage |  |  |  |
| Ⅰ, Ⅱ | 146 (89.6%) | 17(10.4%) | <0.0001 ^b^ |
| Ⅲ, Ⅳ | 76 (45.2%) | 92(54.8%) |  |

^a^Student’s test.

^b^Fisher's exactTest

**Supplementary Table 3. Summary of ChIP-Seq short reads**

| **ChIP sample** | **Sequencer** | **Sequence length** | **Reads (X 10^6^)** | **Mapped Reads (X 10^6^)** | **Mapping%** |
| --- | --- | --- | --- | --- | --- |
| α-Sp1 | AB 5500xl SOLiD | 35 bp | 26.36 | 18.48 | 70.11% |
| α-IgG |  |  | 21.66 | 15.37 | 70.97% |

Total reads from each sample were mapped to the reference human genome (hg19).

**Supplementary Table 4. Genomic distribution of Sp1-bound regions**

| **Regions**  **Counts** | **Promoter*** | **5´-UTR** | **Exon** | **Intron** | **3´-UTR** | **Intergenic**** |
| --- | --- | --- | --- | --- | --- | --- |
| **Sp1-bound** | 3,372 | 1,069 | 4,717 | 48,630 | 2,136 | 62,575 |

ChIP-Seq analysis was done using CLC Genomics Workbench.

*2kb upstream of transcription start site (TSS).

**Excluding promoter, 5´-UTR, exon, intron and 3´-UTR regions.

**Supplementary Table 5. Gene biotype of Sp1-regulated genes**

| **Gene biotype** | **Sp1 ChIP-Seq*** | **Total**** |
| --- | --- | --- |
| Protein coding | 2,655 | 22,836 |
| miRNA | 141 | 3,361 |
| lincRNA | 67 | 7,340 |
| snoRNA | 32 | 1,549 |
| snRNA | 4 | 2,077 |
| rRNA | 1 | 566 |

*The number of genes whose upstream 2 kb region from TSS contains Sp1 ChIP-Seq binding signals.

**Total number of genes within the biotype from Ensembl database (Ensembl Genes 75 - Homo sapiens genes, GRCh37.p13).
